# 3D Bioprinted Scaffolds Containing Mesenchymal Stem/Stromal Lyosecretome: Next Generation Controlled Release Device for Bone Regenerative Medicine

**DOI:** 10.3390/pharmaceutics13040515

**Published:** 2021-04-08

**Authors:** Elia Bari, Franca Scocozza, Sara Perteghella, Marzio Sorlini, Ferdinando Auricchio, Maria Luisa Torre, Michele Conti

**Affiliations:** 1 Department of Drug Sciences, University of Pavia, 27100 Pavia, Italy; elia.bari@unipv.it (E.B.); sara.perteghella@unipv.it (S.P.); 2 Department of Civil Engineering and Architecture, University of Pavia, 27100 Pavia, Italy; franca.scocozza01@universitadipavia.it (F.S.); auricchio@unipv.it (F.A.); michele.conti@unipv.it (M.C.); 3 P4P S.r.l., 27100 Pavia, Italy; 4 PharmaExceed S.r.l., 27100 Pavia, Italy; marzio.sorlini@supsi.ch; 5 SUPSI—Department of Innovative Technologies, Lugano University Centre, 6962 Viganello, Switzerland

**Keywords:** mesenchymal stem cells, MSC secretome, MSC extracellular vesicles, 3D printing, poly(ε-caprolactone), bone tissue engineering, bone regenerative medicine

## Abstract

Three-dimensional printing of poly(ε-caprolactone) (PCL) is a consolidated scaffold manufacturing technique for bone regenerative medicine. Simultaneously, the mesenchymal stem/stromal cell (MSC) secretome is osteoinductive, promoting scaffold colonization by cells, proliferation, and differentiation. The present paper combines 3D-printed PCL scaffolds with lyosecretome, a freeze-dried formulation of MSC secretome, containing proteins and extracellular vesicles (EVs). We designed a lyosecretome 3D-printed scaffold by two loading strategies: (i) MSC secretome adsorption on 3D-printed scaffold and (ii) coprinting of PCL with an alginate-based hydrogel containing MSC secretome (at two alginate concentrations, i.e., 6% or 10% *w*/*v*). A fast release of proteins and EVs (a burst of 75% after 30 min) was observed from scaffolds obtained by absorption loading, while coprinting of PCL and hydrogel, encapsulating lyosecretome, allowed a homogeneous loading of protein and EVs and a controlled slow release. For both loading modes, protein and EV release was governed by diffusion as revealed by the kinetic release study. The secretome’s diffusion is influenced by alginate, its concentration, or its cross-linking modes with protamine due to the higher steric hindrance of the polymer chains. Moreover, it is possible to further slow down protein and EV release by changing the scaffold shape from parallelepiped to cylindrical. In conclusion, it is possible to control the release kinetics of proteins and EVs by changing the composition of the alginate hydrogel, the scaffold’s shape, and hydrogel cross-linking. Such scaffold prototypes for bone regenerative medicine are now available for further testing of safety and efficacy.

## 1. Introduction

In 1993, Langer and Vacanti defined tissue engineering as an interdisciplinary field that applies the principles of engineering and life sciences toward the development of biological substitutes that restore, maintain, or improve tissue function or a whole organ [1]. To this end, three general components are often required: (i) reparative cells/tissues (cell component) that can form a functional matrix, (ii) an appropriate scaffold for transplantation and support, and (iii) bioactive signaling molecules, such as cytokines and growth factors, that will support and choreograph the formation of the desired tissue.

Regarding the cell component, over the years, attention has focused on the use of stem cells, specifically adult stem cells, like mesenchymal stem/stromal cells (MSCs) [2]. The considerable success of MSCs is linked to their ease of isolation and manipulability, as well as the potential for differentiation into various tissues, such as cartilage, bone, fat, muscle, among others [3]. However, 20 years after their discovery, in 2010, Caplan suggested renaming MSCs as “medicinal signaling cells” since their multipotency seemed not to be the only therapeutic aspect [4,5]. The substances secreted by MSCs, and able to modulate resident cell responses, are collectively named secretome, which is composed of free-soluble factors (including cytokines, chemokines, and growth factors) and insoluble nano-/microstructured extracellular vesicles (EVs) [6]. MSC secretome can reproduce, indeed, the therapeutic effects of stem cells themselves, and cell-free therapies should provide numerous advantages compared with cell therapy in terms of safety and technological issues [7,8]. In this respect, our research group already proposed MSC secretome formulated into a standardized and ready-to-use freeze-dried powder, named lyosecretome, and prepared according to production processes compliant with the good manufacturing practice (GMP), thus moving forward to its clinical application [9,10,11,12].

A variety of biocompatible and biodegradable synthetic polymers, such as polylactic acid (PLA), polyglycolic acid (PGA), poly(lactic-co-glycolic acid) (PLGA), and poly(ε-caprolactone) (PCL), have become widespread for the manufacturing of scaffolds. Notably, in the bone regeneration field, PCL is one of the biomaterials widely used thanks to hydrolysis and enzymatic digestion over a tunable period of 2 years, which corresponds to the bone healing range [13]. Moreover, PCL has adequate mechanical strength that makes it resistant to physical, chemical, and mechanical insults [14]. Such polymers can be shaped in any form and size by a variety of traditional preparation techniques, such as electrospinning [15], phase separation [16], freeze drying [17], and self-assembly [18], to name a few examples.

More recently, additive manufacturing technology (e.g., 3D printing and bioprinting) has been employed to fabricate scaffolds with a complex shape and internal porous structure [19,20]. The 3D printing process is generally articulated as follows: (i) a virtual 3D ComputerAided Design (CAD) model is created starting from information collected from patient scans or computer simulations; (ii) based on 3D printing technology, a set of instructions, namely, G-code, is created and exported to the 3D printer, allowing the precise control and construction of the model through a layer-by-layer deposition.

Unfortunately, the in vivo grafting of a 3D-printed scaffold does not always end successfully: sometimes, the cells do not colonize the scaffold, causing tissue necrosis [21,22]. The enrichment of the scaffold with the MSC secretome could promote, on the one hand, the in vitro proliferation and differentiation of cells seeded on the scaffold and, on the other hand, the scaffold colonization when it is implanted in vivo [23,24]. Indeed, the growth factors, cytokines, other proteins, and oligonucleotides of MSC secretome are responsible for transmitting information between cells in the body, stimulating cell proliferation and division, and sustaining new tissue formation [25]. In this regard, the scaffold becomes critical to retain the molecule(s) at the implantation site for a certain time and for its controlled release. Sometimes, a rapid release of signaling molecules is essential to trigger the regeneration mechanism promptly, as recently suggested [26]. However, more often, a slow and controlled release is more appropriate [27].

**Figure 1:**
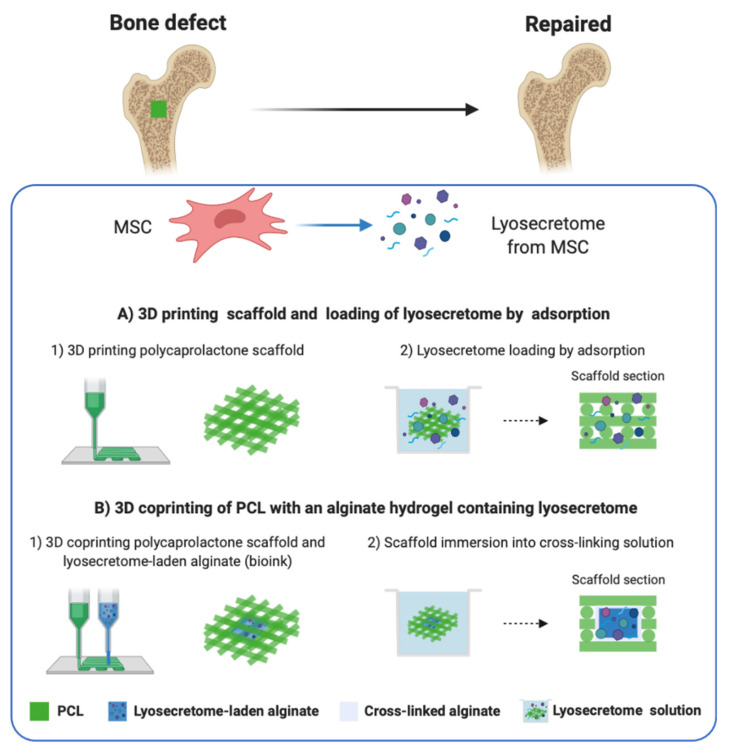
Scheme of the two different strategies investigated in this study: (**A**) 3D printing of the scaffold and subsequent loading of lyosecretome by adsorption. (**B**) 3D coprinting of PCL with an alginate-based hydrogel encapsulating lyosecretome.

To this end, a 3D (bio)printing strategy has already been proposed to manufacture printing scaffolds embedding extracellular vesicles for bone repair and regeneration [28]; however, there are still few studies, and many of them are preliminary. In this context, the present study proposes the design and manufacturing of 3D-printed PCL scaffolds, enriched with lyosecretome, intended for the in vivo controlled release of MCS paracrine factors in bone regenerative medicine. In particular, two different scaffold manufacturing strategies have been proposed as shown in Figure 1: (A) printing of the scaffold and subsequent loading of lyosecretome by adsorption and (B) coprinting [29,30,31] of PCL with an alginate hydrogel containing lyosecretome (in the following referred to as bioink [32]). The first loading mode aims at providing a fast release, while the second one targets a slower release. To this end, alginate was chosen thanks to its ability to form hydrogels when hydrated and achieve a sustained release of secretome proteins and lipids. Finally, scaffolds with different geometries have been designed and characterized in the morphology and release kinetics of secretome proteins and lipids.

## 2. Materials and Methods

### 2.1. Materials

Culture media, trypsin-EDTA, and antibiotics used for cell cultures were purchased from Euroclone (Milan, Italy). A commercial platelet lysate kit (PL) was obtained from Carlo Erba Reagents (Milan, Italy). Acetone, bovine serum albumin (BSA), calcium chloride (CaCl_2_), Lutrol^®^ F127, mannitol, phosphatidylcholine (PC), protamine, sodium alginate (low viscosity, 100–300 cP, 2% *w*/*v*, 25 °C, M/G ratio: 1.96, molecular size: 9500 Da), and sodium chloride were purchased from Sigma-Aldrich (Milan, Italy). PCL pellets (50 kDa) were purchased from Cellink AB (Gothenburg, Sweden). Unless specified otherwise, all reagents were of analytical grade.

### 2.2. Lyosecretome Preparation and Characterization

Freeze-dried MSC secretome (named lyosecretome) was prepared and characterized according to previously reported procedures with slight modifications [9]. It is worth noting that the employed isolation procedures are compatible with the current good manufacturing practice (GMP).

#### 2.2.1. MSC Culture and Secretome Collection

Adipose-derived MSCs (AD-MSCs) were harvested from adipose tissues [33,34] collected from patients undergoing abdominoplasty after informed consent (ASST Grande Ospedale Metropolitano Niguarda, Milan, Ref. 12 November 2009). Donors with septicemia or extensive infections, type B and C hepatitis, HIV, Creutzfeldt–Jakob disease, syphilis, malignant tumors, viral diseases, or unknown neurological diseases were excluded. MSCs were seeded into flasks (10,000 cells/cm^2^) at 37 °C and 5% CO_2_ and cultured in complete culture medium (DMEM/F12 minimal medium plus 5% *v*/*v* PL, plus 1% *v*/*v* penicillin/streptomycin and 1% *v*/*v* amphotericin B) until passage 3. Then, secretome release was induced: MSCs were cultured in DMEM/F12 without platelet lysate for 48 h; conditioned media were collected after 9, 24, 33, and 48 h and pooled. MSCs were detached with trypsin-EDTA and tested to evaluate cell viability, the concordance with all the requirements needed for clinical use in terms of identity (according to the International Society for Cellular Therapy [35]) and sterility (according to Eu. Ph. 9.0, 2.6.27). 

#### 2.2.2. MSC Secretome Ultrafiltration and Lyophilization

Conditioned media were centrifuged at 3500× *g* for 10 min to eliminate cell debris and apoptotic bodies; supernatants were collected and ultrafiltered by tangential flow filtration (KrosFlo^®^ Research 2i system, Spectrum Laboratories, Milan, Italy) equipped with a filtration module with a molecular weight cut-off (MWCO) of 5 kDa (Spectrum Laboratories, Milan, Italy) and with a superficial area of 235 cm^2^. Both free-soluble proteins and EVs produced by MSCs were retained. In detail, samples were at first concentrated to 0.5 × 10^6^ cell equivalents per mL (calculated by dividing the total cell number and the mL of concentrated and purified supernatant) and then diafiltered using sterilized ultrapure water. The transmembrane pressure did not exceed 10 psi, and the shear rate of the feed stream was maintained between 2000 and 6000 s^−1^, as prescribed by the manufacturer. Mannitol was dissolved into concentrated and purified secretome (final concentration of 0.5% *w*/*v*); the resulting solution was frozen at −80 °C and freeze-dried (Christ Epsilon 2-16D LSCplus) at 8 × 10^−1^ mbar and −50 °C for 72 h. The obtained lyosecretome was stored at −20 °C until use (6 months). Each mg of lyosecretome corresponds to 0.1 × 10^6^ cell equivalents (calculated by dividing the total cell number used for the production and the obtained milligrams of lyosecretome).

#### 2.2.3. Lyosecretome Protein and Lipid Content

The BCA Protein Assay Kit from Thermo Fisher Scientific (Milan, Italy) was used to assess lyosecretome total protein content. The working reagent solution was prepared according to the manufacturer’s instructions and then added to each sample (or standard) at a 1:1 ratio. After incubation at 37 °C for 2 h, the absorbance was measured at 562 nm with a microplate reader (Synergy HT, BioTek, Swindon, United Kingdom). The protein concentration was extrapolated from a concentration vs. absorbance plot obtained from standard protein solutions (BSA), using a third-degree polynomial equation, with *R*^2^ = 0.99. Lipids were quantified by the Nile Red assay as previously reported and validated [9]. A Nile Red stock solution in acetone (3.14 M) was prepared and stored at 4 °C, avoiding light exposure until use. The stock solution was diluted 100 × in phosphate-buffered saline (PBS) before use, and 10 µL of it was incubated with 90 µL of samples; after 5 min, the relative fluorescence was measured by Synergy HT at fixed wavelengths (530/25 excitation and 645/40 emission). The lipid concentration was extrapolated from a concentration vs. fluorescence plot obtained from standard lipid solutions (PC), using a third-degree polynomial equation, with *R*^2^ = 0.99. Results are reported as µg of proteins/lipids per mg of lyosecretome (mean values ± standard deviation, three independent samples from the same lyosecretome production batch).

### 2.3. PCL Scaffold Preparation and Lyosecretome Loading

Two different strategies were investigated: (i) printing of the scaffold and then loading of lyosecretome or (ii) coprinting of PCL with a lyosecretome-laden alginate hydrogel suspension. For the second strategy, two different alginate concentrations were tested (6% and 10% *w*/*v*). Alginate hydrogel was cross-linked with 2% *w*/*v* CaCl_2_ solution and double cross-linked with 2% *w*/*v* CaCl_2_ and 5% *w*/*v* protamine.

In both cases, the PCL scaffolds were 3D-printed with the Cellink INKREDIBLE+, a pneumatic extrusion-based 3D bioprinter with dual heated printheads (PHs) and a UV LED curing system (365, 405 nm). The 3D bioprinter is equipped with a HEPA 13 filter and a positive air pressure to ensure a sterile environment during printing. The operational variables of the 3D bioprinter are summarized in Table 1. The process starts from a 3D CAD model that is translated into printing instructions (the G-code) by a slicing software, which generates the coordinates of the PH in the *XY*-plane and controls material extrusion. In particular, the 3D CAD model of scaffold structures was designed using the SolidWorks^®^ software (Dassault Systèmes SolidWorks Corporation, Waltham, MA, USA). Then the model was sliced using Slic3r, an open-source slicing software.

An aluminum cartridge with a 0.5 mm metal nozzle was used for 3D-printing PCL pellets. In detail, the cartridge was filled with PCL pellets, placed into the first PH (PH1), and heated at 90 °C for 30 min before printing. The printing temperature was kept constant at 90 °C, and the pressure was set at 85 kPa.

#### 2.3.1. Printing of PCL Scaffolds and Lyosecretome Loading (PCL)

**Figure 2:**
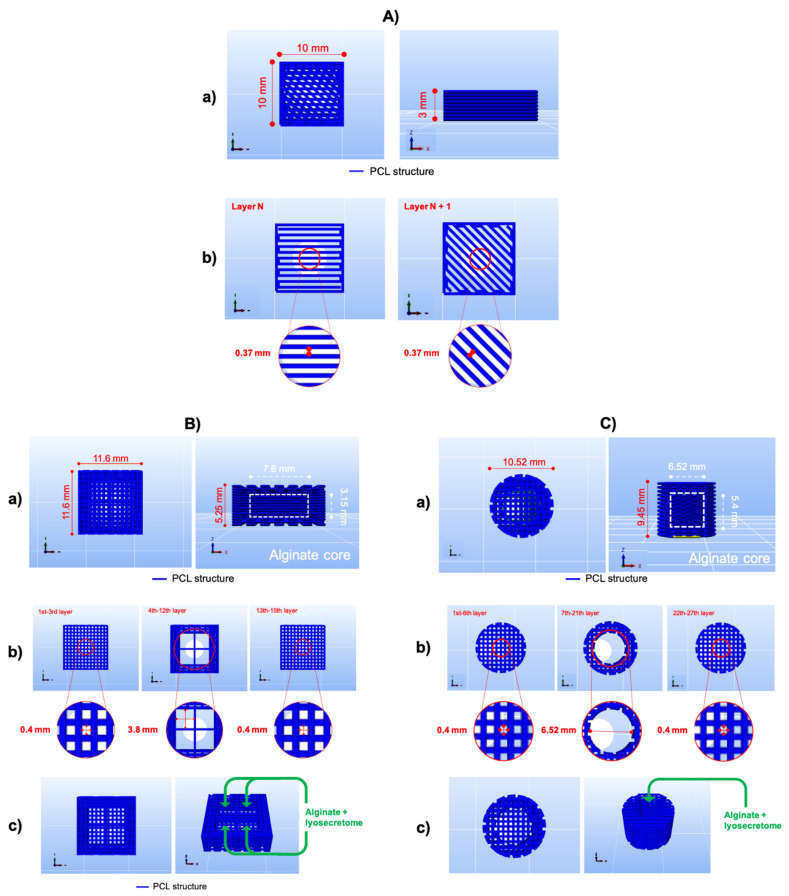
Scaffold geometry and dimensions for the two different strategies investigated. (**A**) PCL scaffold 3D-printed and subsequentially loaded with lyosecretome; planar and sectional view of the total scaffold (**a**) and planar view of the scaffold layers (**b**). (**B**) Coprinting parallelepiped- and (**C**) cylindrical-shaped scaffolds with a “soft heart” of lyosecretome-laden alginate (i.e., bioink). Planar and sectional views of the total scaffold (**a**), planar view of the scaffold layers (**b**), and well into bioink was extruded (**c**).

A 3D CAD model of a parallelepiped-shaped structure (10 × 10 × 3 mm, Figure 2Aa) was designed and sliced, getting a structure made of nine layers of 0.35 mm height with one perimeter in each. Two consecutive layers were shifted by 45°. Layer infill was set at 45%, corresponding to 0.37 mm of fiber distance (i.e., the distance between two adjacent fibers) (Figure 2Ab). Printing speed was set at 45 mm/min. The G-code was created, and PCL porous scaffolds were 3D-printed on a Petri dish.

For the lyosecretome loading, scaffolds were placed in a 24-Multiwell, covered with an aqueous solution of lyosecretome (15% *w*/*v*), Lutrol^®^ F127, and NaCl (0.1% *w*/*v* each) for 1.5 h at 4 °C, frozen at −80 °C and freeze-dried (Christ Epsilon 2-16D LSCplus) at 8 × 10^−1^ mbar and −50 °C for 72 h. Lutrol^®^ F127 was added to increase the scaffold’s wettability (and thus allow homogeneous loading), while NaCl was added to reduce mannitol crystallization during the freeze-drying process [36]. For the loading determination, PCL scaffolds were dispersed in deionized water under magnetic stirring for 96 h. Proteins and lipids were dosed into the final solution according to the procedures reported in Section 2.2.3. Each experiment was performed in triplicate.

#### 2.3.2. 3D Coprinting of PCL Scaffolds with Alginate Hydrogels (PCL-Alg6, PCL-Alg10, PCL-Alg10p, cPCL-Alg6, cPCL-Alg10, and cPCL-Alg10p)

Porous PCL scaffolds (11.6 × 11.6 × 5.25 mm) with a “soft heart” of alginate, laden with lyosecretome to form the bioink, were 3D-coprinted. In particular, the porous external structure was printed with PCL to create one or more wells to contain 200 μL of bioink solution (Figure 2B,C).

This study considered two different scaffold geometries: a parallelepiped-shaped geometry (11.6 × 11.6 × 5.25 mm, Figure 2Ba) and a cylindrical one (d: 10.52 mm, h: 9.45 mm, Figure 2Ca). The parallelepiped-shaped structure was made of 15 layers of 0.35 mm height, each with a fiber distance fixed at 0.4 mm oriented at 90° (Figure 2Bb). The alginate solution was extruded into four little wells of 3.8 mm by the side and 3.15 mm by height (Figure 2Bc, Supplementary Movie S1). The cylinder-shaped structure was 3D-coprinted for studying the release kinetics, and it was made of 27 layers of 0.35 mm height each, and the fiber distance was fixed at 0.4 mm oriented at 90° (Figure 2Cb). In this case, the bioink solution was printed into only one internal well of 6.52 mm by diameter and 5.4 by height (Figure 2Cc, Supplementary Movie S2).

**Figure 3:**
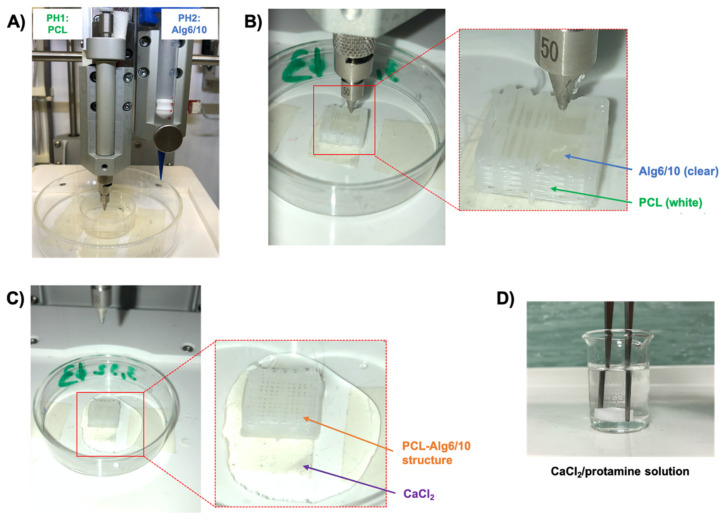
3D coprinting setup and process of the parallelepiped-shaped scaffold. (**A**) PCL scaffold and lyosecretome-laden alginate (i.e., bioink) were 3D-printed using the PH1 and PH2, respectively. (**B**) The first part of the PCL scaffold was 3D-printed, forming the four little wells in which bioink was extruded. The last part of the structure was 3D-printed to cover the wells and create the “soft heart.” (**C**) The scaffold was first covered with a CaCl_2_ solution for 5 min. (**D**) Finally, the scaffold was immersed into a CaCl_2_ solution, and it was gently shaken for 5 min to allow the solution to go beyond the PCL scaffold and reach the bioink. For double cross-linking, the scaffold was later immersed into a protamine solution and stirred gently for a further 5 min.

Both PHs were used for coprinting PCL and bioink: the first one (PH1) for printing PCL and the second one (PH2) for printing bioink. Lyosecretome powder was dispersed in the alginate solution at a final concentration of 12.5 mg/mL to form the bioink; then a plastic cartridge was filled with the bioink, and then it was placed into PH2 (Figure 3A). The printing pressure was set at 15 and 20 kPa for 6% and 10% *w*/*v* alginate, respectively. PCL-Alg6 scaffolds were prepared with a 6% *w*/*v* alginate solution, while PCL-Alg10 scaffolds were prepared with a 10% *w*/*v* alginate solution. We performed both single and double cross-linking of alginate concentrations. In the first case, the polymer was cross-linked with a 2% *w*/*v* CaCl_2_ solution; in the second case, the polymer was cross-linked first with 2% *w*/*v* CaCl_2_ and subsequently with a 5% *w*/*v* protamine solution.

The 3D coprinting process involves the following steps: (i) the first part of the PCL scaffold forming the well was printed; (ii) a printing stop was programmed to moisten the printed structure with a small amount of CaCl_2_ and avoid alginate dispersion; (iii) 200 µL bioink was extruded into the well; (iv) the last part of the PCL structure was 3D-printed to cover the well and to create the “soft heart” (Figure 3B); and (v) the scaffold was first covered with a 2% *w*/*v* CaCl_2_ solution for 1 min (Figure 3C), and then it was immersed into a CaCl_2_ solution, and it was gently shaken for 5 min to allow the solution to go beyond the PCL scaffold and reach the hydrogel (Figure 3D). Similarly, for double cross-linking, the scaffold was moved from a CaCl_2_ solution to a protamine solution and stirred gently for a further 5 min.

For the loading determination, the scaffolds were dispersed in deionized water under magnetic stirring for 240 h. Proteins and lipids were dosed into the final solution according to the procedures reported in Section 2.2.3. Each experiment was performed in triplicate. 

#### 2.3.3. Morphological and Structural Characterizations by Scanning Electron Microscopy (SEM)

Samples for scanning electron microscopy (SEM) analysis were previously sectioned with a steel blade to observe their inner structure. The samples were subsequently attached to SEM stubs with conductive adhesive carbon tape and metal-coated with 10 nm chromium using a high-vacuum Quorum Q150T ES Plus sputtering system. SEM imaging was performed with High-Resolution SEM Zeiss Gemini 500 FEG-SEM (Zeiss, Germany) or SEM MIRA3 (Tescan, Brno, Czech Republic) operating with an acceleration voltage of 5 kV. The samples were analyzed before and after the drug release studies. Furthermore, SEM images were used to extract sample dimensions (e.g., layer height, fiber distance) using the ImageJ software (Rasband, W.S., ImageJ, U.S. National Institutes of Health, Bethesda, MD, USA).

#### 2.3.4. Drug Release Studies

Three-dimensional printed scaffolds loaded with lyosecretome were immersed into pH 7.2 phosphate-buffered saline (PBS, USP). At fixed time intervals, predetermined volumes of the released PBS were withdrawn and replaced by an equivalent amount of fresh PBS. The lipid and protein released were determined by the Nile Red and BCA assays, respectively, as previously reported in Section 2.2.3. Each analysis was performed in triplicate. The cumulative amount of released proteins/lipids was calculated as a percentage using Equation (1):

Cumulative amount of drug released (%) = C_i_/C_0_ × 100 (1)

where C_i_ is the amount of the proteins/lipids released at a definite time interval, and C_0_ is the loaded protein/lipid amount.

#### 2.3.5. Drug Release Kinetic Study

The in vitro drug release data was interpolated using different kinetic models, as reported below.

Higuchi

*F*(*t*) = *k* × *t*^0.5^ (2)

*F*(*t*) = 100 × (1 − C × exp ^(−*k* × *t*)^) (3)

where *F*(*t*) is the amount of drug dissolved at time *t* and *k* is the release constant. Equation (3) was reproduced from (Equation (2.12) from [37]).

Peppas–Sahlin

*F*(*t*) = *k*_1_ × *t^m^* + *k*_2_ × *t*^(2 × *m*)^ (4)

where *F*(*t*) is the amount of drug dissolved at time *t*, *k*_1_ is the diffusion constant, *k*_2_ is the erosion constant, and *m* is the diffusional exponent, indicative of the drug release mechanism.

Ritger–Peppas

*F*(*t*) = *k* × *t^n^* (5)

where *F*(*t*) is the amount of drug dissolved at time *t*, *k* is the release constant, and *n* is the release exponent, indicative of the drug release mechanism.

Zero-order

*F*(*t*) = *k* × *t* (6)

where *F*(*t*) is the amount of drug released in time *t*, and *k* is the release constant.

Korsmeyer–Peppas

*F*(*t*) = *k_KP_* × *t^n^* × Q_0_ (7)

where *F*(*t*) is the amount of drug released at time t; *k_KP_* is the release constant; *n* is the release exponent, indicative of the drug release mechanism; and Q_0_ is the initial amount of drug.

### 2.4. Statistical Analysis

Raw data were processed using Statgraphics XVII (Statpoint Technologies, Inc., Warrenton, VA, USA). A generalized linear analysis of variance model (ANOVA) was used, followed by Fisher’s least significant difference (LSD) procedure to estimate the differences between the means. In detail, drug loading results were analyzed considering the batch as a fixed factor and the drug loading as the response variable. The statistical significance was set at *p* < 0.05.

## 3. Results and Discussion

The use of MSC secretome combined with tissue engineering strategies may result in a new generation of osteoinductive scaffolds, which can improve the speed and quality of new bone formation. In this work, MSC secretome was prepared according to GMP-compliant procedures, which allowed us to formulate it into a standardized and ready off-the-shelf product, named lyosecretome. In detail, MSC secretome was isolated and purified from cell culture supernatants by ultrafiltration and then freeze-dried [9]. Mannitol was used as a cryoprotectant to maintain the EV layer’s integrity and avoid protein destabilization induced by the freezing and drying stresses generated during the lyophilization process [38]. MSCs used for the lyosecretome preparation fulfilled the International Society for Cellular Therapy (ISCT) criteria [35]. In detail, at the end of serum starvation, MSCs were positive for CD90, CD73, and CD105 and negative for CD45, and were able to differentiate, upon stimulation, into chondrocyte, adipocyte, and osteocyte (data not shown). Lyosecretome contained 8.32 ± 0.238 µg of proteins and 0.789 ± 0.0190 µg of lipids per mg of powder (mean values ± standard deviation, three independent replicates from the same lyosecretome batch).

We investigated two strategies for 3D-printing PCL scaffolds used for the MSC secretome delivery in the bone regeneration applications (Figure 1). The first strategy consists of the printing of the scaffold and the subsequent loading of MSC secretome by adsorption. Such a method was investigated as it can be considered the simplest, straightforward, and cost-effective [39]. Figure S1 in the Supplementary Materials section reports the structural and morphological characterizations of PCL scaffolds performed by SEM. The PCL scaffold structure is visible, and the surface of PCL fibers appears smooth and uniform. Table 2 shows a comparison between the 3D reference model and 3D-printed PCL scaffold dimensions. The mean and standard deviation values indicate that the printed model dimensions are near the target one unless there are errors due to the printing process.

Next, lyosecretome was loaded onto PCL scaffolds by immersion and subsequent freeze-drying, resulting in a drug coating over the material’s surfaces. Lutrol^®^ F127 (poloxamer 407) and NaCl were added to the lyosecretome solution as excipients. Poloxamer 407 is widely used to enhance the solubilization of poorly water-soluble drugs, and it appears to be more effective than polyol or polysorbate [40]. Here it was used to increase the scaffold’s wettability and increase the liquid penetration into the inner core, allowing a homogeneous loading of secretome proteins and lipids. It is worth noting that poloxamer 407 also promotes stabilization of proteins, and thus, together with mannitol, it can prevent/reduce the propensity for secretome–peptide unfolding [41,42]. Preliminary investigations revealed that mannitol crystallizes during the freeze-drying step, leading to the formation of needlelike crystals emerging from the surface of the scaffold (data not shown). Therefore, the formulation was optimized by adding NaCl. In their work, Telang et al. reported that salts, specifically NaCl, inhibited mannitol crystallization more effectively than surfactants, cyclodextrins, polymers, and aldols [36]. Indeed, the addition of NaCl prevented mannitol crystallization at the surface of the scaffold during the freeze-drying step.

**Figure 4:**
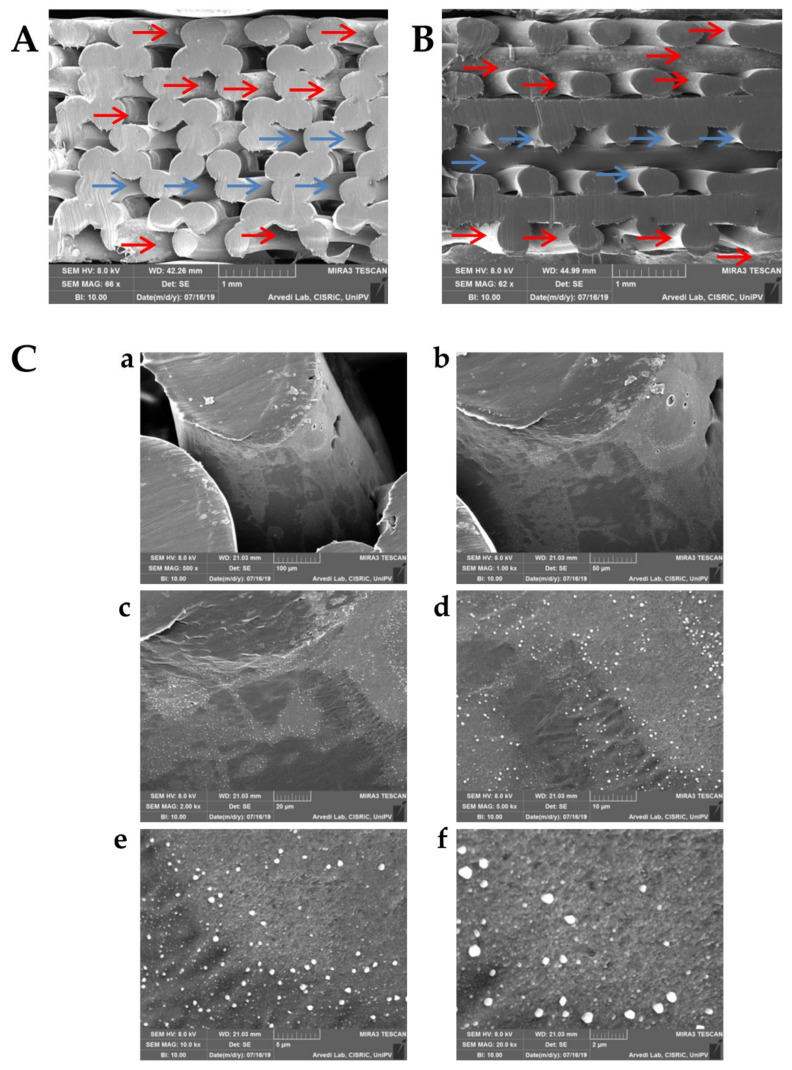
SEM morphological and structural characterizations of 3D-printed PCL scaffolds loaded with lyosecretome, two vertical sections (**A**,**B**). Presence (red arrows) or absence (blue arrows) of material deposition on PCL fibers is indicated. Increasing magnifications of the material deposited on PCL fibers (**C**, magnifications **a**—**f**). Scale bar: 1 mm for (**A**) and (**B**); 100 µm for (**a**); 50 µm for (**b**); 20 µm for (**c**); 10 µm for (**d**); 5 µm for (**e**); 2 µm for (**f**).

The average loading of PCL scaffolds was 720.29 ± 118.418 µg for proteins and 50.10 ± 20.925 µg for lipids (*n* = 3). The high batch-to-batch variability indicates that the loading process by this method should be improved to allow a homogeneous loading. SEM analyses also confirmed such results. In Figure 4, it is possible to distinguish some clean areas in the scaffold (they appear smooth and uniform, blue arrows) from those covered with lyosecretome (Figure 4A, red arrows). Specifically, the internal region of the scaffold is difficultly reached by the secretome solution; therefore, no material deposition on the surface of the PCL fibers is observed (Figure 4B, blue arrows). Figure 4C confirms that lyosecretome adhesion on the external area of the PCL scaffold occurs, even with a nonhomogeneous distribution of proteins and lipids (Figure 4C, magnifications a–d). From higher magnifications (Figure 4Ce,f), it is possible to distinguish NaCl (which appears as white cubic crystals) from other rounded structures, with a smooth surface and 200 nm size, attributable to the other components of the formulation (mannitol) and the vesicular component of the secretome.

**Figure 5:**
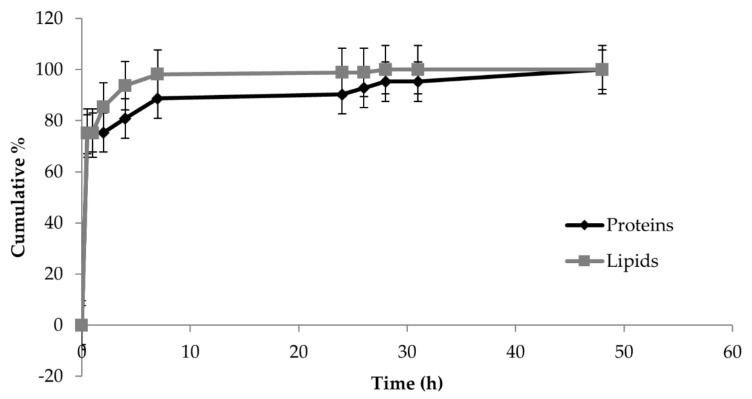
In vitro protein and lipid release profiles from PCL scaffolds immersed in pH 7.2 phosphate-buffered saline (PBS) at room temperature. Multifactor ANOVA, mean values ± least significant difference (LSD), *n* = 3.

Protein and lipid release profiles are shown in Figure 5. The cumulative release from PCL scaffolds showed an initial burst release, in 30 min, of 75% for both proteins and lipids. Ninety percent of proteins were released after 24 h, while, at the same time, 98% of lipids were released. This rapid release was an expected result, as the components of the formulation (mannitol, poloxamer 407, and NaCl) are hydrophilic compounds, which easily recall the PBS into the scaffold and dissolve. At the end of the release studies, SEM investigation revealed that the PCL fibers appeared smooth and uniform, indicating the complete release of secretome from the scaffold (data not shown).

The second strategy proposes the coprinting of PCL and hydrogel encapsulating lyosecretome to both improve the homogeneous loading of proteins/lipids and slow down their release. To this end, lyosecretome was incorporated into an alginate hydrogel to form the bioink and then coprinted into a porous PCL scaffold. The coprinting process allows for extruding simultaneously two different biomaterials (thermoplastic and hydrogel) to create a hybrid structure useful as a delivery system. Two alginate concentrations were considered (6% and 10% *w*/*v*), obtaining PCL-Alg6 and PCL-Alg10 scaffolds, respectively. At the end of the printing process, PCL-Alg6 and PCL-Alg10 scaffolds were cross-linked with calcium chloride (CaCl_2_). PCL-Alg10p scaffolds were then prepared, double cross-linking PCL-Alg10 with CaCl_2_ and protamine. The average loading of each scaffold is reported in Table 3.

It has to be noted that higher proteins were detected for PCL-Alg10p scaffolds because the protamine used for cross-linking alginate remained a residue. Overall, a more homogeneous loading for both proteins and lipids was achieved with respect to the first method. 

**Figure 6:**
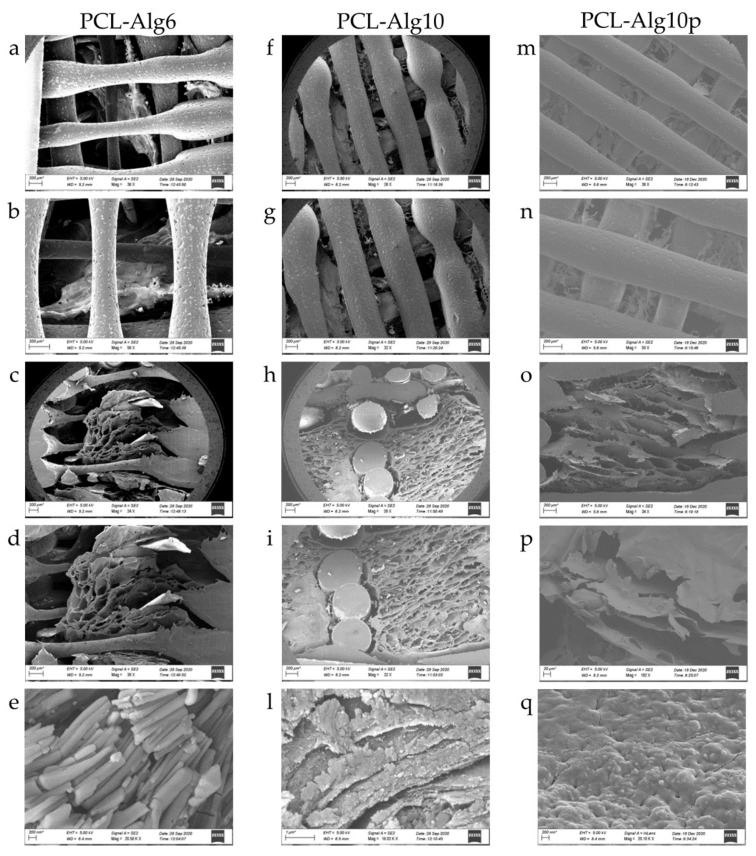
Morphological investigation by SEM of PCL-Alg6, PCL-Alg10, and PCL-Alg10p scaffolds before drug release studies (**a–d**, **f–i** and **m–p**). Higher magnifications of the bioink surface are also reported (**e**,**l**,**q**) to highlight the vesicular component of the lyosecretome. Scale bar: 200 µm for (**a–i**, **m–o** and **q**); 1 µm for (**l**); 20 µm for (**p**).

Figure 6 reports the structural and morphological characterizations of scaffolds. Observing the scaffolds above, it is possible to discern the bioink core among the PCL fibers (Figure 6, magnifications a, b, f, g, m, and n). PCL fibers appear irregular because they are the last layers after the wells and, therefore, do not have much support to adhere to. The scaffolds were then sectioned to better expose the alginate contained in the inner structure. Bioink appears with a wide mesh net structure, typical of lyophilized products. Specifically, for scaffolds loaded with higher concentrations of alginate, the mesh structure appears denser (Figure 6, magnifications h and i vs. magnifications c and d). A similar structure was also observed for the scaffolds cross-linked with protamine, even if the alginate layers appeared more compact (Figure 6, magnifications o and p). At higher magnifications, bioink’s surface, for both of the concentrations investigated, appears with a rough surface, characterized by many elongated relief structures (Figure 6, magnifications e, l). For scaffolds cross-linked with protamine, bioink appeared more compact and with a porous structure (Figure 6, magnification q). At the highest magnifications, it is also possible to distinguish spherical relief structures, approximately 200 nm in diameter, attributable to the vesicular component of the secretome (Figure 6, magnifications l and q).

Table 4 shows a comparison between the 3D reference model and 3D-printed PCL scaffold dimensions. The mean and standard deviation values of the 3D-printed scaffolds are 0.33 ± 0.03 mm for layer height and 0.38 ± 0.05 mm for fiber distance. The layer height and fiber distance values of the printed model are comparable to those of the target one unless there are errors due to the printing process.

**Figure 7:**
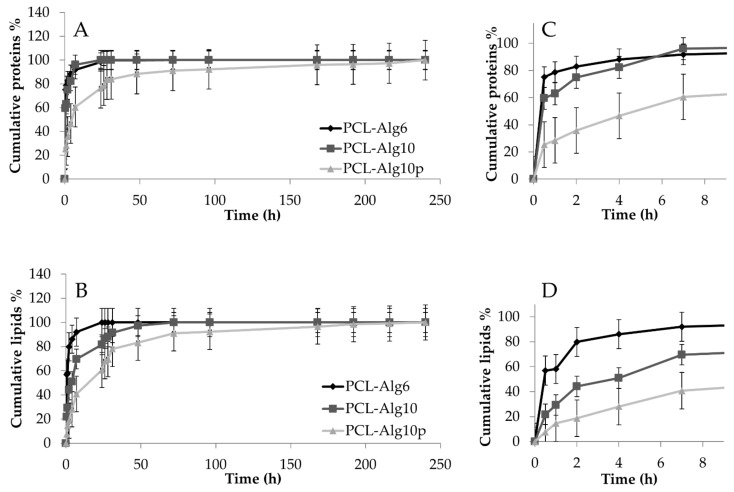
In vitro protein (**A**) and lipid (**B**) release profiles from PCL-Alg6, PCL-Alg10, and PCL-Alg10p scaffolds immersed in pH 7.2 PBS at room temperature. On the right, for each graph, an enlargement of the first 8 h of release is reported (**C**, **D** for PCL-Alg6 and PCL-Alg10, respectively). Multifactor ANOVA, mean values ± least significant difference (LSD), *n* = 3.

Drug release data revealed that the coprinting of bioink and PCL effectively slowed down the release of lyosecretome proteins and lipids (Figure 7). For the PCL-Alg6 scaffold, the protein release profile was a superimposable one of the PCL scaffold: 75% of proteins were released in the first 30 min, and after 24 h, 98% of proteins were released. Conversely, lipid release was significantly slowed down. In detail, PCL-Alg6 scaffolds released 57% of lipids after 30 min (vs. 75% of PCL scaffold, *p* < 0.05). Then, lipid release gradually increased over time, reaching 86% after 4 h and 100% after 24 h. By increasing the alginate concentration, both protein and lipid releases were further slowed down. PCL-Alg10 scaffolds released 60% of proteins after 30 min (vs. 75% of both PCL and PCL-Alg6 scaffolds, *p* < 0.05); protein release was gradually increased until reaching 100% after 24 h. After 30 min, only 21% of lipids were released (vs. 57% of PCL-Alg6 and 75% of PCL, *p* < 0.05); after 24 h, not all lipids were released (81%), and a complete release was reached after 48 h. In addition to alginate’s effect, protamine cross-linking further slows down the release of proteins and lipids. In detail, for PCL-Alg10p scaffolds, only 25% of proteins were released after 30 min (*p* < 0.05 compared with PCL-Alg6 and PCL-Alg10), and the release gradually increased, reaching 100% after 240 h (vs. 24 h for both PCL-Alg6 and PCL-Alg10, *p* < 0.05). A similar behavior was observed for lipids: PCL-Alg10p scaffolds released only 7% of lipids after 30 min and then 60% after 24 h (both values are significantly lower than those for PCL-Alg6 and PCL-Alg10, *p* < 0.001). One hundred percent of lipids were released after 240 h.

The different release behavior of proteins and lipids, namely, EVs, into the alginate hydrogels is expected. Alginate is a hydrophilic compound, and therefore, it can facilitate the absorption and diffusion of water (by recalling it) inside the scaffold. Alginate dissolves into the recalled PBS, and a hydrogel is formed. According to a concentration gradient, proteins diffuse into such hydrogel, from the inner region with greater concentration to the outer less concentrated one. At low alginate concentration (6% *w*/*v*), polymer chains offer a low steric hindrance, which does not sufficiently slow down the diffusion of proteins. By increasing the alginate concentration, the steric hindrance is increased as well, and thus proteins diffuse slowly through polymer chains. On the other hand, nanostructured or microstructured particulate structures (i.e., EVs) diffuse less quickly in a hydrophilic environment, as happens to the alginate hydrogel. The cross-linking of alginate with protamine led to a more compact structure, as reported in Figure 6, within which proteins and lipids diffuse slowly [43].

At the end of the release studies, SEM investigations were performed to assess whether all the alginate core was released. From Figure S2 reported in the Supplementary Materials section, it is possible to observe that most of the alginate was released from the scaffolds. Only small portions of material remained in the lower part of the scaffolds (red arrows in Figure S2). This is more evident for PCL-Alg10 scaffolds, as the higher concentration of alginate slowed down its dissolution process.

Next, the influence of the scaffold geometry on protein and lipid release was investigated. To this end, cylindrical scaffolds were printed and loaded with alginate at two different concentrations (6% and 10% *w*/*v*), obtaining cPCL-Alg6 and cPCL-Alg10 scaffolds cross-linked with CaCl_2_. cPCL-Alg10p was then prepared, double cross-linking cPCL-Alg10 with CaCl_2_ and protamine. Table 5 reports the average protein and lipid loading of each scaffold.

Even in this case, it has to be noted that higher proteins were detected for cPCL-Alg10p scaffolds because the protamine used for cross-linking alginate remained a residue. Overall, a more homogeneous loading for both proteins and lipids was achieved with respect to the first method.

**Figure 8:**
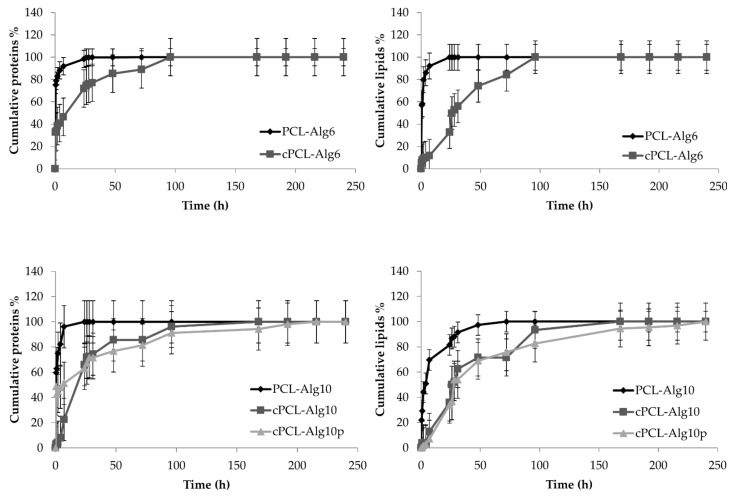
In vitro protein and lipid release profiles from PCL-Alg6, PCL-Alg10, PCL-Alg10p, cPCL-Alg6p, and cPCL-Alg10p scaffolds immersed in pH 7.2 PBS at room temperature. Multifactor ANOVA, mean values ± least significant difference (LSD), *n* = 3.

By keeping the alginate concentration of the hydrogel constant, the cylindrical shape significantly slowed proteins and lipids’ release (Figure 8). In detail, the burst effect was reduced considerably: cPCL-Alg6 scaffolds released 32% of proteins after 30 min vs. 75% of PCL-Alg6 (*p* < 0.05) and 5% of lipids, namely, EVs, vs. 57% of PCL-Alg6 (*p* < 0.05). Similarly, cPCL-Alg10 scaffolds released 2% of proteins after 30 min vs. 60% of PCL-Alg10 (*p* < 0.05) and less than 1% of lipids, namely, EVs, vs. 22% of PCL-Alg10 (*p* < 0.05). A gradual release of proteins and lipids was sustained for both cPCL-Alg6 and cPCL-Alg10 scaffolds, and the complete release of lyosecretome was achieved after 96 and 168 h, respectively. Even in this case, cross-linking with protamine further reduced EV release from the cPCL-Alg10p scaffolds (*p* < 0.05 compared with PCL-Alg10 and cPCL-Alg10), while the effect on proteins was less evident. This can be explained considering that protamine may remain a residue and dosed together with lyosecretome proteins. Therefore, it can be expected that lyosecretome proteins also slowly released after protamine cross-linking.

To gain a deeper understanding of the release mechanisms, the drug release data were further processed by elaborating the kinetic model of the release systems. Higuchi, Peppas–Sahlin, Ritger–Peppas, and zero-order are among the most employed models and describe the release of the encapsulated molecules as a function of time, thus providing information about the exact mass transport mechanisms involved in the drug release. Table 6 lists the in vitro release model results fitting for the Ritger–Peppas and Korsmeyer–Peppas models for all the scaffolds prepared (PCL, PCL-Alg6, PCL-Alg10, PCL-Alg10p, cPCL-Alg6, cPCL-Alg10, and cPCL-Alg10p). The results of the other kinetic models are reported in the Supplementary Materials section (Table S1).

Protein and lipid release from all the scaffolds followed the Ritger–Peppas and Korsmeyer–Peppas models, for which proper fitting values were calculated. Indeed, *R*^2^ values were above 0.90 except for cPCL-Alg6 scaffolds, for which *R*^2^ values were about 0.4. These models help study drug release from polymeric systems when the release mechanism is unknown or when more than one type of drug release phenomenon is involved [44,45]. Specifically, it can be seen as a generalization of the superposition of two independent drug transport mechanisms: relaxation and diffusion. Depending on the value of *n* that better adjusts to the drug’s release profile, it is possible to distinguish between two drug release models, the Fickian model (case I) and the non-Fickian models (case II, anomalous case and super case II). However, the interpretation of the release exponent (*n*) is valid only for drug release systems with a defined geometry (planar as thin films, cylindrical or spherical). Thus, it does not apply to the geometry of our scaffolds. Moreover, for the cylindrical scaffold, the alginate hydrogel core (which controlled the release) is cylindrical, whereas for the parallelepiped scaffold, it is parallelepiped-shaped.

For PCL scaffolds, while PBS diffuses inside the scaffold, it solubilizes the lyosecretome deposited on the PCL fibers. Then proteins and lipids diffuse into the solvent around PCL fibers according to a concentration gradient, from the inner region with greater concentration to the outer less concentrated one. For the PCL-ALg6 and PCL-Alg10 scaffolds, the alginate molecule will undergo almost immediate hydration to create a hydrocolloid layer of high viscosity. This makes up a diffusion barrier, decreasing the migration of both proteins and lipids. Indeed, comparing the *k*-values obtained from the Ritger–Peppas and Korsmeyer–Peppas equations, it is possible to observe that PCL scaffolds have the highest *k*-values for both proteins (79.99) and lipids (95.38). For PCL-Alg6, only the *k*-values for lipids decreased (70.22, *p* < 0.05 vs. PCL), while a significant reduction was observed for the *k*-values of both proteins and lipids in the PCL-Alg10 scaffolds (68.7 and 35.57, respectively, *p* < 0.05). For the PCL-Alg10p scaffolds, *k*-values were further reduced to 21.9 for proteins and 34.09 for lipids (*p* < 0.05 compared with the PCL-Alg6 and PCL-Alg10 scaffolds). Overall, this confirms the release data: diffusion of proteins and lipids is reduced when increasing the alginate concentration or when cross-linking with protamine due to the steric hindrance of the polymer chains, and this becomes particularly evident for lipids, namely, EVs, as they diffuse less quickly in the hydrophilic alginate hydrogel. In the case of cylindrical scaffolds, the protein/lipid release mechanism remains the same, but low *k*-values are obtained, as expected due to the slow release. In detail, for cPCL-Alg6, *k*-values were reduced to 29.03 for proteins and 3.949 for lipids (*p* < 0.05 compared with PCL-Alg6); for cPCL-Alg10, *k*-values were reduced to 39.99 for proteins and 4.533 for lipids (*p* < 0.05 compared with PCL-Alg10). Even for cPCL-Alg10p, cross-linking with protamine reduced *k*-values for proteins (*p* < 0.05 compared with cPCL-Alg10p) and for lipids (*p* < 0.05 compared with cPCL-Alg10). Finally, good fitting was also observed for the Higuchi equation, confirming the role of diffusion in protein and lipid release from scaffolds (see Table S1).

Overall, the change in scaffold geometry did not modify the drug release mechanism from the hydrogel alginate. As for cPCL-Alg6, cPCL-Alg10, and cPCL-Alg10p, the best fittings were still for the Ritger–Peppas and Korsmeyer–Peppas equations. The PCL geometry may, therefore, explain the different release profiles [46,47,48,49]. According to Chew et al. [48], the scaffold-surface-area-to-volume ratio (SVR) is a factor that plays a role in determining the characteristics of polymer material degradation, which in turn influence molecule release. The greater the SVR, the greater the degradation, increasing the kinetics release. Indeed, a polymeric matrix with higher SVR has a greater surface to allow encapsulated agents to spread more quickly [49]. In light of these considerations, in our case, the SVR of the parallelepiped-shaped scaffold is equal to 0.72, while the SVR of the cylindrical scaffold is equal to 0.59. Therefore, the SVR of the parallelepiped-shaped scaffold is greater than the SVR of the cylinder-shaped scaffold, confirming faster release kinetics. Therefore, we speculate that the release profile could be controlled by varying the PCL scaffold geometry and fibers’ distance and orientation [47].

Although the use of 3D bioprinting to enrich scaffolds with MSC EVs has been already proposed, we are convinced that the current approach has a significant novelty level. We are introducing two key aspects in the scaffold manufacture to enhance the control of lyosecretome release: the use of a coprinting technique and the alginate. Coprinting allows the simultaneously controlled deposition of different biomaterial inks and bioinks (e.g., PCL and lyosecretome-laden alginate, respectively) during the scaffold manufacturing; we combined PCL to ensure the structural stability of the scaffold that has to sustain mechanical loads when implanted and alginate encapsulating lyosecretome. As we have shown, the combination of the PCL design (i.e., shape and porosity) with the alginate composition and cross-linking increases the degree of freedom to control the lyosecretome release. Such an approach is different from the one proposed by Diomede et al. [23], who evaluated the in vitro and in vivo abilities of the bone defect regeneration effect of 3D-printed polylactide (PLA) scaffolds coated with polyethyleneimine (PEI), enriched with human gingival MSC (hGMSC), and complexed with electric vehicles and engineered electric vehicles. Diomede et al. are manufacturing the scaffolds and then coating them; this approach can potentially impair the coating’s homogeneous distribution, especially in its inner core, when the scaffold is complex or the porosity is high. Moreover, as we have shown, this approach provides a burst release that can potentially limit the desired medium- or long-term efficacy of MSCs’ paracrine factors.

It is worth noting that the use of 3D-printed hydrogels to embed MSC-derived exosomes has already been proposed for cartilage regeneration by Chang et al. [24]. They applied a desktop-stereolithography (SLA) technology for high-resolution and photo-cross-linked biomaterial printing. In particular, they proposed a bioink composed of MSC-derived exosomes, decellularized cartilage ECM, and gelatin methacrylate (GelMA) hydrogel. Such a bioink cannot have sufficient mechanical stability to hold the mechanical stimuli required for bone repair, while our solution is to ensure mechanical stability and release control.

## 4. Conclusions

MSC secretome combination with 3D-printed biomaterial inks and bioinks [32] is an attractive approach in regenerative medicine to obtain next-generation osteoinductive scaffolds, improving the speed and quality of new bone formation. In this paper, two different strategies were designed to load lyosecretome—a freeze-dried and formulated MSC secretome—onto 3D-printed PCL scaffolds. The first strategy consisted of impregnating scaffolds with secretome and was investigated as it is the simplest, straightforward, and cost-effective. With this method, a fast release of the secretome from the scaffold was achieved: after 30 min, 75% of both proteins and lipids, namely, EVs. Nevertheless, high batch-to-batch loading variability indicates that this process can be improved. The second strategy consisted of the coprinting of PCL and hydrogel encapsulating lyosecretome (PCL-Alg6 and PCL-Alg10). PCL-ALg10p scaffolds were also prepared, cross-linking alginate with protamine. A homogeneous loading of proteins/lipids was achieved, and their release was slowed down. Drug-release kinetic studies revealed that for both PCL and PCL-Alg6/PCL-Alg10/PCL-Alg10p scaffolds, the protein/lipid release is governed by diffusion. Specifically, diffusion is reduced with alginate, increasing alginate concentration, or when cross-linking with protamine due to the steric hindrance of the polymer chains. By changing the scaffold shape from parallelepiped to cylindrical, it is possible to further slow down protein and EV release.

In conclusion, the proof of concept for manufacturing a cutting-edge controlled-release 3D-bioprinted scaffold containing lyosecretome has been provided. By changing the composition of the alginate hydrogel, shape of the scaffold, and cross-linking with protamine, it is possible to control the release kinetics of proteins and EVs. Prototypes are now available for bone regenerative medicine safety and efficacy tests.

## Figures and Tables

**Table 1:** Summary of 3D printer operational variables used for the study.

3D Printer Operational Variable	PCL	Lyosecretome-Laden Alginate	
		6% (*w*/*v*)	10% (*w*/*v*)
Extrusion pressure (kPa)	85	10	20
Conical nozzle diameter (mm)	0.5	0.41	
Printing speed (mm/min)	45	600	
Printing temperature	90 °C	Room temperature	

**Table 2:** Scaffold 3D printing and subsequent loading of MSC secretome by adsorption. Comparison between dimensions of the 3D model and 3D-printed PCL scaffolds (measure from SEM images, *n* = 6).

3D Scaffold	Layer Height (mm)	Fiber Distance (mm)
Reference model	0.35	0.37
Printed (mean ± std. dev)	0.35 ± 0.02	0.34 ± 0.06

**Table 3:** Protein and lipid loading for PCL-Alg6, PCL-Alg10, and PCL-Alg10p scaffolds. Mean values ± standard deviation (*n* = 3).

Scaffold	Proteins (µg)	Lipids (µg)
PCL-Alg6	188.68 ± 18.386	25.00 ± 7.042
PCL-Alg10	104.52 ± 16.977	55.10 ± 11.588
PCL-Alg10p	538.63 ± 11.729	131.63 ± 15.715

**Table 4:** Coprinting of PCL with an alginate hydrogel containing MSC secretome. Comparison between dimensions of the 3D model and 3D-printed PCL scaffolds (measure from SEM images, *n* = 6).

3D Scaffold	Layer Height (mm)	Fibers Distance (mm)
Reference model	0.35	0.4
Printed (mean ± std. dev)	0.33 ± 0.03	0.38 ± 0.05

**Table 5:** Protein and lipid loading for cPCL-Alg6, cPCL-Alg10, and cPCL-Alg10p scaffolds. Mean values ± standard deviation (*n* = 3).

Scaffold	Proteins (µg)	Lipids (µg)
cPCL-Alg6	218.75 ± 243.383	69.82 ± 76.169
cPCL-Alg10	342.66 ± 39.920	85.77 ± 0.0521
cPCL-Alg10p	543.97 ± 105.892	37.86 ± 28.954

**Table 6:** Results of in vitro release model fitting for PCL, PCL-Alg6, PCL-Alg10, PCL-Alg10p, cPCL-Alg6, cPCL-Alg10, and cPCL-Alg10p. Kinetic elaborations were performed on release data obtained from at least three independent experiments for each batch. ~ indicates that the analysis performed was “ambiguous”; therefore, the fit does not nail down the values of all the parameters, and 95% confidence bounds cannot be reported. These latter data were not considered in the interpretation of results.

Model	Equation	Sample	Proteins/Lipids	Coefficients (95% Confidence Bounds)	Sum of Squares	*R* ^2^	Degrees of Freedom	SE
Ritger–Peppas	*F*(*t*) = *k* × *t^n^*	PCL	Proteins	*k* = 79.99 (77.02, 82.96) *n* = 0.05101 (0.03735, 0.06487)	716.4	0.9698	31	*k* 1.449 *n* 0.006697
			Lipids	*k* = 95.38 (89.26, 101.5) *n* = 0.0322 (0.008344, 0.05660)	3145	0.9026	31	*k* 3.044 *n* 0.01204
		PCL-Alg6	Proteins	*k* = 84.66 (80.32, 89.01) *n* = 0.05194 (0.03319, 0.07108)	1558	0.9434	31	*k* 2.136 *n* 0.00932
			Lipids	*k* = 70.22 (64.70, 75.79) *n* = 0.1097 (0.08268, 0.1378)	1107	0.9442	20	*k* 2.708 *n* 0.01351
		PCL-Alg10	Proteins	*k* = 68.7 (63.49, 75.79) *n* = 0.1166 (0.09065, 0.1435)	2393	0.9225	31	*k* 2.607 *n* 0.01322
			Lipids	*k* = 35.57 (32.07, 39.14) *n* = 0.2775 (0.2470, 0.3098)	1301	0.9622	31	*k* 1.782 *n* 0.01585
		PCL-Alg10p	Proteins	*k* = 21.9 (14.7, 28.7) *n* = 0.1835 (0.1157, 0.2611)	492,356	0.6315	31	*k* 34.81 *n* 0.03748
			Lipids	*k* = 34.09 (18.84, 53.25) *n* = 0.2648 (0.1645, 0.3881)	46,372	0.5897	31	*k* 9.229 *n* 0.05876
		cPCL-ALg6	Proteins	*k* = 29.03 (3.229, 88.22) *n* = 0.4657 (0.1563, 0.9889)	271,193	0.3611	31	*k* 23.17 *n* 0.2043
			Lipids	*k* = 3.949 (0.2132, 17.73) *n* = 0.6505 (0.2522, 1.328)	23,253	0.4061	31	*k* 4.294 *n* 0.2682
		cPCL-Alg10	Proteins	*k* = 39.99 (27.29, 55.59) *n* = 0.4998 (0.4152, 0.5948)	35,392	0.9223	31	*k* 7.771 *n* 0.04934
			Lipids	*k* = 4.533 (3.162, 6.269) *n* = 0.6616 (0.5811, 0.7492)	882.3	0.9628	31	*k* 0.8106 *n* 0.04402
		cPCL-Alg10p	Proteins	*k* = 21.8 (19.6, 24.8) *n* = 0.1667 (0.1403, 0.1945)	58,586	0.9184	31	*k* 12.32 *n* 0.01299
			Lipids	*k* = 2.099 (0.5318, 5.270) *n* = 0.54 (0.3499, 0.8064)	3687	0.6421	31	*k* 1.241 *n* 0.1183
Korsmeyer–Peppas	*F*(*t*) = *k_KP_* × *t^n^* × Q_0_	PCL	Proteins	*k_KP_* = 79.99 (77.02, 82.96) *n* = 0.05101 (0.03735, 0.06487)	716.4	0.9698	31	*k_KP_* 1.449 *n* 0.006697
			Lipids	*k_KP_* = 95.38 (89.26, 101.5) *n* = 0.0322 (0.008344, 0.05660)	3145	0.9026	31	*k_KP_* 3.044 *n* 0.01024
		PCL-Alg6	Proteins	*k_KP_* = 84.66 (80.32, 89.01) *n* = 0.05194 (0.03319, 0.07108)	1558	0.9434	31	*k_KP_* 2.136 *n* 0.00932
			Lipids	*k_KP_* = 70.22 (64.70, 75.79) *n* = 0.1097 (0.08268, 0.1378)	1107	0.9442	20	*k_KP_* 2.708 *n* 0.01351
		PCL-Alg10	Proteins	*k_KP_* = 68.7 (63.49, 73.95) *n* = 0.1166 (0.09065, 0.1435)	2393	0.9225	31	*k_KP_* 2.607 *n* 0.01322
			Lipids	*k_KP_* = 35.57 (32.07, 39.14) *n* = 0.2775 (0.2470, 0.3098)	1301	0.9622	31	*k_KP_* 1.782 *n* 0.01585
		PCL-Alg10p	Proteins	*k_KP_* = 21.9 (14.7, 28.7) *n* = 0.1835 (0.1157, 0.2611)	492,356	0.6315	31	*k_KP_* 34.81 *n* 0.03748
			Lipids	*k_KP_* = 34.09 (18.84, 53.25) *n* = 0.2648 (0.1645, 0.3881)	46,372	0.5897	31	*k_KP_* 9.229 *n* 0.05876
		cPCL-Alg6	Proteins	*k_KP_* = 29.03 (3.229, 88.22) *n* = 0.4657 (0.1563, 0.9889)	271,193	0.3611	31	*k_KP_* 23.17 *n* 0.2043
			Lipids	*k_KP_* = 3.949 (0.2132, 17.73) *n* = 0.6505 (0.2522, 1.328)	23,253	0.4061	31	*k_KP_* 4.294 *n* 0.2682
		cPCL-Alg10	Proteins	*k_KP_* = 39.99 (27.29, 55.59) *n* = 0.4998 (0.4152, 0.5948)	35,392	0.9223	31	*k_KP_* 7.771 *n* 0.04934
			Lipids	*k_KP_* = 4.533 (3.162, 6.269) *n* = 0.6616 (0.5811, 0.7492)	882.3	0.9628	31	*k_KP_* 0.8106 *n* 0.04402
		cPCL-Alg10p	Proteins	*k_KP_* = 21.8 (19.6, 24.8) *n* = 0.1667 (0.1403, 0.1945)	58,586	0.9184	31	*k_KP_* 12.32 *n* 0.01299
			Lipids	*k_KP_* = 2.099 (0.5318, 5.270) *n* = 0.54 (0.3499, 0.8064)	3687	0.6421	31	*k_KP_* 1.241 *n* 0.1183

## Data Availability

The data presented in this study are contained within the article.

## Supplementary Materials

The following are available online at https://www.mdpi.com/article/10.3390/pharmaceutics13040515/s1: Figure S1: Morphology and structure characterizations of 3D-printed PCL scaffolds for the first strategy. SEM images were taken at increasing magnifications (26×, 120×, and 500×). Figure S2: Morphological investigation by SEM of PCL-Alg6, PCL-Alg10, and PCL-Alg10p scaffolds after drug release studies. Red arrows indicate the small portions of alginate remaining after release. Figure S3: Lyosecretome alginate core position inside the PCL scaffold structure. For the parallelepiped-shaped structure (A), the distance from the alginate core to the outside is not the same in XYZ directions. In the cylindrical-shaped structure (B), there is the same distance from the alginate core to the outside in all XYZ directions. Video S 1: Coprinting of alginate hydrogel containing lyosecretome into parallelepiped PCL scaffolds. Video S2: Coprinting of alginate hydrogel containing lyosecretome into cylindrical PCL scaffolds. Table S1: Results of in vitro release model fitting for PCL, PCL-Alg6, PCL-Alg10, PCL-Alg10p, cPCL-Alg6, cPCL-Alg10, and cPCL-Alg10p. Kinetic elaborations were performed on release data obtained from at least three independent experiments for each batch. ~ indicates that the analysis performed was “ambiguous”; therefore, the fit does not nail down the values of all the parameters, and 95% confidence bounds cannot be reported. These latter data were not considered in the interpretation of results.

## References

[1] Langer R., Vacanti J. (1993). Tissue engineering. Science.

[2] Gomez-Salazar M., Gonzalez-Galofre Z.N., Casamitjana J., Crisan M., James A.W., Péault B. (2020). Five Decades Later, Are Mesenchymal Stem Cells Still Relevant?. Front. Bioeng. Biotechnol..

[3] Rosenbaum A.J., Grande D.A., Dines J.S. (2008). The use of mesenchymal stem cells in tissue engineering: A global assessment. Organogenesis.

[4] Caplan A.I. (2010). What’s in a Name?. Tissue Eng. Part A.

[5] Gnecchi M., He H., Liang O.D., Melo L.G., Morello F., Mu H., Noiseux N., Zhang L., Pratt R.E., Ingwall J.S. (2005). Paracrine action accounts for marked protection of ischemic heart by Akt-modified mesenchymal stem cells. Nat. Med..

[6] Crivelli B., Chlapanidas T., Perteghella S., Lucarelli E., Pascucci L., Brini A.T., Ferrero I., Marazzi M., Pessina A., Torre M.L. (2017). Mesenchymal stem/stromal cell extracellular vesicles: From active principle to next generation drug delivery system. J. Control. Release.

[7] Bari E., Ferrarotti I., Torre M.L., Corsico A.G., Perteghella S. (2019). Mesenchymal stem/stromal cell secretome for lung regeneration: The long way through “pharmaceuticalization” for the best formulation. J. Control. Release.

[8] Bari E., Ferrarotti I., Saracino L., Perteghella S., Torre M.L., Corsico A.G. (2020). Mesenchymal Stromal Cell Secretome for Severe COVID-19 Infections: Premises for the Therapeutic Use. Cells.

[9] Bari E., Perteghella S., Di Silvestre D., Sorlini M., Catenacci L., Sorrenti M., Marrubini G., Rossi R., Tripodo G., Mauri P. (2018). Pilot Production of Mesenchymal Stem/Stromal Freeze-Dried Secretome for Cell-Free Regenerative Nanomedicine: A Validated GMP-Compliant Process. Cells.

[10] Bari E., Perteghella S., Catenacci L., Sorlini M., Croce S., Mantelli M., Avanzini M.A., Sorrenti M., Torre M.L. (2019). Freeze-dried and GMP-compliant pharmaceuticals containing exosomes for acellular mesenchymal stromal cell immunomodulant therapy. Nanomedicine.

[11] Bari E., Ferrarotti I., Di Silvestre D., Grisoli P., Barzon V., Balderacchi A., Torre M.L., Rossi R., Mauri P., Corsico A.G. (2019). Adipose Mesenchymal Extracellular Vesicles as Alpha-1-Antitrypsin Physiological Delivery Systems for Lung Regeneration. Cells.

[12] Perteghella S., Bari E., Chlapanidas T., Sorlini M., De Girolamo L., Perucca Orfei C., Viganò M., Torre M.L. (2016). Process for Isolating and Lyophilizing Extracellular Vesicles.

[13] Fan D., Staufer U., Accardo A. (2019). Engineered 3D Polymer and Hydrogel Microenvironments for Cell Culture Applications. Bioengineering.

[14] Dwivedi R., Kumar S., Pandey R., Mahajan A., Nandana D., Katti D.S., Mehrotra D. (2020). Polycaprolactone as biomaterial for bone scaffolds: Review of literature. J. Oral Biol. Craniofacial Res..

[15] Jun I., Han H.-S., Edwards J.R., Jeon H. (2018). Electrospun Fibrous Scaffolds for Tissue Engineering: Viewpoints on Architecture and Fabrication. Int. J. Mol. Sci..

[16] Sofokleous P., Chin M.H., Day R. (2018). Phase-separation technologies for 3D scaffold engineering. Functional 3D Tissue Engineering Scaffolds—Materials, Technologies and Applications.

[17] Fereshteh Z. (2018). Freeze-drying technologies for 3D scaffold engineering. Functional 3D Tissue Engineering Scaffolds—Materials, Technologies and Applications.

[18] Peck M., Dusserre N., McAllister T.N., L’Heureux N. (2011). Tissue engineering by self-assembly. Mater. Today.

[19] Muwaffak Z., Goyanes A., Clark V., Basit A.W., Hilton S.T., Gaisford S. (2017). Patient-specific 3D scanned and 3D printed antimicrobial polycaprolactone wound dressings. Int. J. Pharm..

[20] Soufivand A.A., Abolfathi N., Hashemi A., Lee S.J. (2020). The effect of 3D printing on the morphological and mechanical properties of polycaprolactone filament and scaffold. Polym. Adv. Technol..

[21] Huang R.-L., Kobayashi E., Liu K., Li Q. (2016). Bone Graft Prefabrication Following the In Vivo Bioreactor Principle. EBioMedicine.

[22] Alaribe F.N., Manoto S.L., Motaung S.C. (2016). Scaffolds from biomaterials: Advantages and limitations in bone and tissue engineering. Biologia.

[23] Diomede F., Gugliandolo A., Cardelli P., Merciaro I., Ettorre V., Traini T., Bedini R., Scionti D., Bramanti A., Nanci A. (2018). Three-dimensional printed PLA scaffold and human gingival stem cell-derived extracellular vesicles: A new tool for bone defect repair. Stem Cell Res. Ther..

[24] Chen P., Zheng L., Wang Y., Tao M., Xie Z., Xia C., Gu C., Chen J., Qiu P., Mei S. (2019). Desktop-stereolithography 3D printing of a radially oriented extracellular matrix/mesenchymal stem cell exosome bioink for osteochondral defect regeneration. Theranostics.

[25] Presen D.M., Traweger A., Gimona M., Redl H. (2019). Mesenchymal Stromal Cell-Based Bone Regeneration Therapies: From Cell Transplantation and Tissue Engineering to Therapeutic Secretomes and Extracellular Vesicles. Front. Bioeng. Biotechnol..

[26] Bari E., Di Silvestre D., Mastracci L., Grillo F., Grisoli P., Marrubini G., Nardini M., Mastrogiacomo M., Sorlini M., Rossi R. (2020). GMP-compliant sponge-like dressing containing MSC lyo-secretome: Proteomic network of healing in a murine wound model. Eur. J. Pharm. Biopharm..

[27] Lee K., Silva E.A., Mooney D.J. (2011). Growth factor delivery-based tissue engineering: General approaches and a review of recent developments. J. R. Soc. Interface.

[28] Holkar K., Vaidya A., Pethe P., Kale V., Ingavle G. (2020). Biomaterials and extracellular vesicles in cell-free therapy for bone repair and regeneration: Future line of treatment in regenerative medicine. Materialia.

[29] MacCurdy R., Katzschmann R., Kim Y., Rus D. Printable Hydraulics: A Method for Fabricating Robots by 3D Co-Printing Solids and Liquids. Proceedings of the IEEE International Conference on Robotics and Automation (ICRA).

[30] Kang H.-W., Lee S.J., Ko I.K., Kengla C., Yoo J.J., Atala A. (2016). A 3D bioprinting system to produce human-scale tissue constructs with structural integrity. Nat. Biotechnol..

[31] Samavedi S., Joy N. (2017). 3D printing for the development of in vitro cancer models. Curr. Opin. Biomed. Eng..

[32] Groll J., Burdick J.A., Cho D.-W., Derby B., Gelinsky M., Heilshorn S.C., Jüngst T., Malda J., Mironov V.A., Nakayama K. (2019). A definition of bioinks and their distinction from biomaterial inks. Biofabrication.

[33] Faustini M., Bucco M., Chlapanidas T., Lucconi G., Marazzi M., Tosca M.C., Gaetani P., Klinger M., Villani S., Ferretti V.V. (2010). Nonexpanded Mesenchymal Stem Cells for Regenerative Medicine: Yield in Stromal Vascular Fraction from Adipose Tissues. Tissue Eng. Part C Methods.

[34] Gaetani P., Torre M.L., Klinger M., Faustini M., Crovato F., Bucco M., Marazzi M., Chlapanidas T., Levi D., Tancioni F. (2008). Adipose-Derived Stem Cell Therapy for Intervertebral Disc Regeneration: AnIn VitroReconstructed Tissue in Alginate Capsules. Tissue Eng. Part A.

[35] Dominici M., Le Blanc K., Mueller I., Slaper-Cortenbach I., Marini F.C., Krause D.S., Deans R.J., Keating A., Prockop D.J., Horwitz E.M. (2006). Minimal criteria for defining multipotent mesenchymal stromal cells. The International Society for Cellular Therapy position statement. Cytotherapy.

[36] Telang C., Yu L., Suryanarayanan R. (2003). Effective inhibition of mannitol crystallization in frozen solutions by sodium chloride. Pharm. Res..

[37] Caccavo D. (2019). An overview on the mathematical modeling of hydrogels’ behavior for drug delivery systems. Int. J. Pharm..

[38] Chen C., Han D., Cai C., Tang X. (2010). An overview of liposome lyophilization and its future potential. J. Control. Release.

[39] Costa P.F. (2015). Bone Tissue Engineering Drug Delivery. Curr. Mol. Biol. Rep..

[40] Dumortier G., Grossiord J.L., Agnely F., Chaumeil J.C. (2006). A Review of Poloxamer 407 Pharmaceutical and Pharmacological Characteristics. Pharm. Res..

[41] Wang P.L., Johnston T.P. (1993). Enhanced stability of two model proteins in an agitated solution environment using poloxamer 407. J. Parenter. Sci. Technol..

[42] Katakam M., Banga A.K. (1997). Use of Poloxamer Polymers to Stabilize Recombinant Human Growth Hormone Against Various Processing Stresses. Pharm. Dev. Technol..

[43] Vigo D., Faustini M., Torre M.L., Pecile A., Villani S., Asti A., Norberti R., Maggi L., Conte U., Cremonesi F. (2002). Boar semen controlled-delivery system: Morphological investigation and in vitro fertilization test. Reprod. Fertil. Dev..

[44] Peppas N.A., Narasimhan B. (2014). Mathematical models in drug delivery: How modeling has shaped the way we design new drug delivery systems. J. Control. Release.

[45] Peppas N.A. (1985). Analysis of Fickian and non-Fickian drug release from polymers. Pharm. Acta Helv..

[46] Tamjid E., Bohlouli M., Mohammadi S., Alipour H., Nikkhah M. (2020). Sustainable drug release from highly porous and architecturally engineered composite scaffolds prepared by 3D printing. J. Biomed. Mater. Res. Part A.

[47] Yilgor P., Yilmaz G., Onal M.B., Solmaz I., Gundogdu S., Keskil S., Sousa R.A., Reis R.L., Hasirci N., Hasirci V. (2013). Anin vivostudy on the effect of scaffold geometry and growth factor release on the healing of bone defects. J. Tissue Eng. Regen. Med..

[48] Chew S.A., Arriaga M.A., Hinojosa V.A. (2016). Effects of surface area to volume ratio of PLGA scaffolds with different architectures on scaffold degradation characteristics and drug release kinetics. J. Biomed. Mater. Res. Part A.

[49] Panyam J., Dali M.M., Sahoo S.K., Ma W., Chakravarthi S.S., Amidon G.L., Levy R.J., Labhasetwar V. (2003). Polymer degradation and in vitro release of a model protein from poly(d,l-lactide-co-glycolide) nano- and microparticles. J. Control. Release.

