# Supplementary material for: 3D Bioprinted Scaffolds Containing Mesenchymal Stem/Stromal Lyosecretome: Next Generation Controlled Release Device for Bone Regenerative Medicine
Source: Pharmaceutics. 2021 Apr 8;13(4):515. doi: 10.3390/pharmaceutics13040515 (PMC8070453; doi:10.3390/pharmaceutics13040515)
Supplement: Supplementary file 1 [file pharmaceutics-13-00515-s001.zip › Supplementary materials/pharmaceutics-1143483 - supplementary material for XML .docx]

Supplementary Materials: 3D Bioprinted Scaffolds Containing Mesenchymal Stem/Stromal Lyosecretome: Next Generation Controlled Release Device for Bone Regenerative Medicine

Elia Bari, Franca Scocozza, Sara Perteghella, Marzio Sorlini, Ferdinando Auricchio, Maria Luisa Torre and Michele Conti

| **Publisher’s Note:** MDPI stays neutral with regard to jurisdictional claims in published maps and institutional affiliations.  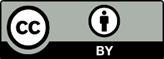  **Copyright:** © 2020 by the authors. Submitted for possible open access publication under the terms and conditions of the Creative Commons Attribution (CC BY) license (http://creativecommons.org/licenses/by/4.0/). |
| --- |


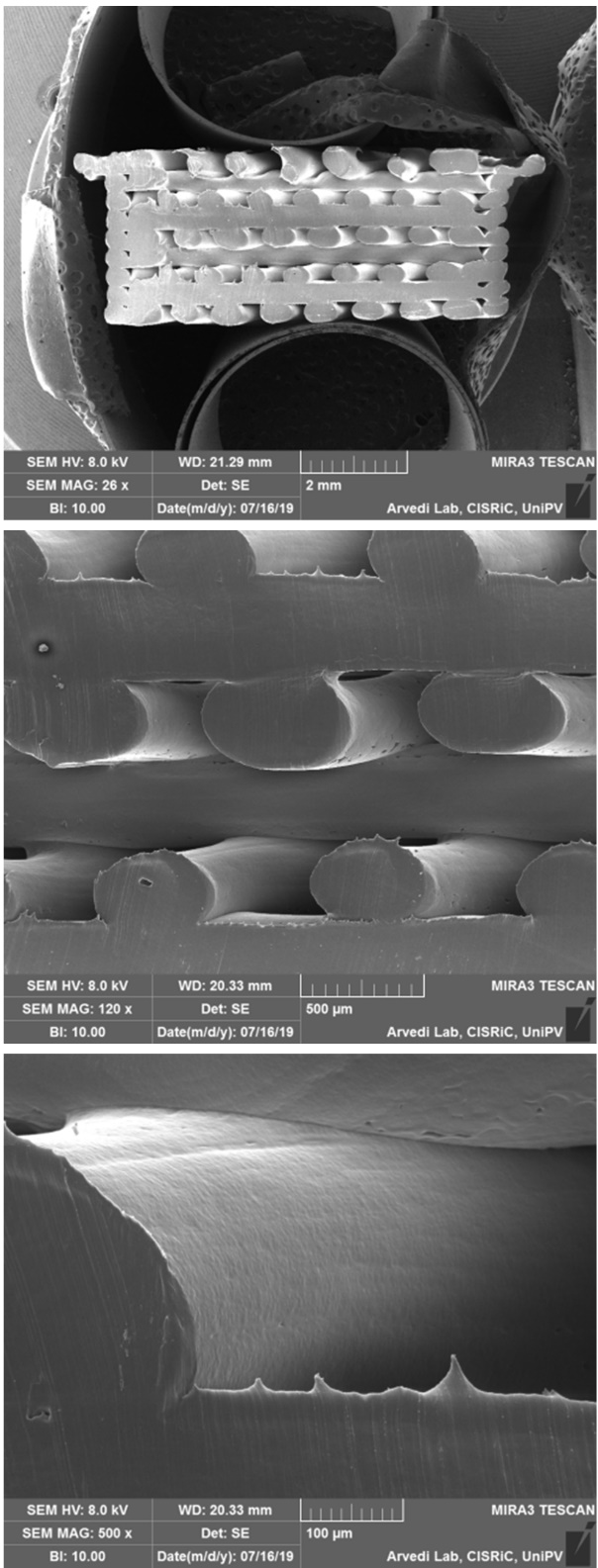


**Figure A1.** Morphology and structure characterization of 3D printed PCL scaffolds for the first strategy. SEM images were taken at increasing magnifications (26 ×,120 × and 500 ×).


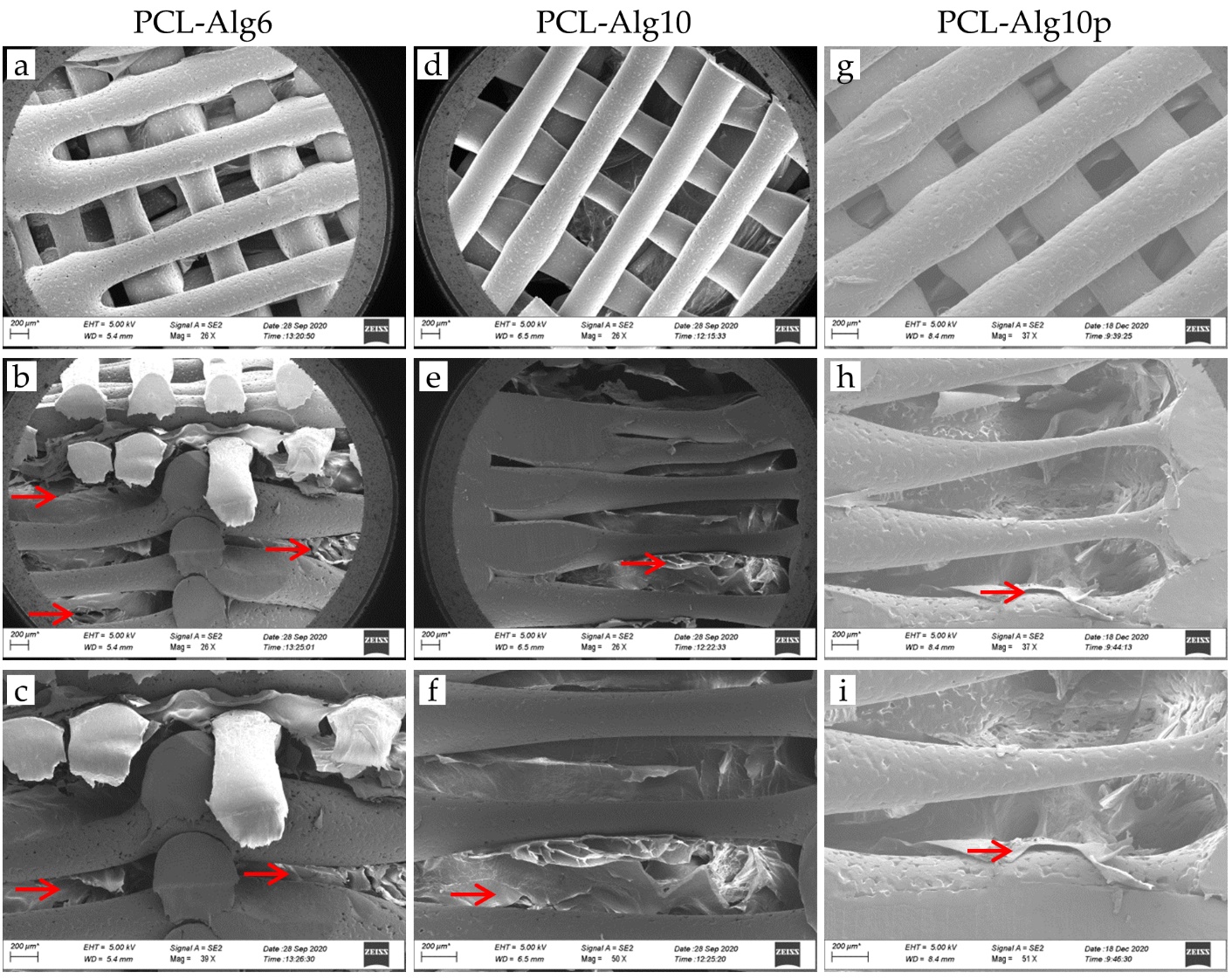


**Figure S2.** Morphological investigation by SEM of PCL-Alg6 (**a—c**), PCL-Alg10 (**d—f**) and PCL-Alg10p (**g—i**) scaffolds after drug release studies. Red arrows indicate the small portions of alginate remained after release.

**Table 1.** Results of in vitro release model fitting for PCL, PCL-Alg6, PCL-Alg10, PCL-Alg10p, cPCL-Alg6, cPCL-Alg10 and cPCL-Alg10p. Kinetic elaborations were performed on release data obtained from at least three independent experiments for each batch. ~ indicates that the analysis performed was “ambiguous”; therefore, the fit does not nail down the values of all the parameters, and 95% confidence bounds cannot be reported. These latter data were not considered in the interpretation of results.

| **Model** | **Equation** | **Sample** | **Proteins/Lipids** | **Coefficients (95% Confidence Bounds)** | **Sum of Squares** | **R^2^** | **Degrees of Freedom** | **SE** |
| --- | --- | --- | --- | --- | --- | --- | --- | --- |
| Higuchi | *F*(*t*) = *k* × *t*^0.5^ | PCL | Proteins | *k* = 19.32  (15.84, 22.79) | 47926 | −1.02 | 32 | 1.706 |
|  |  |  | Lipids | *k* = 21.6  (17.22, 25.97) | 75927 | −1.352 | 32 | 2.147 |
|  |  | PCL-Alg6 | Proteins | *k* = 20.44  (16.69, 24.18) | 55787 | −1.027 | 32 | 1.841 |
|  |  |  | Lipids | *k* = 20.11  (16.22, 24.00) | 25161 | −0.2692 | 21 | 1.869 |
|  |  | PCL-Alg10 | Proteins | *k* = 20.11  (17.08, 23.13) | 36336 | −0.1774 | 32 | 1.486 |
|  |  |  | Lipids | *k* = 17.25  (15.92, 18.58) | 7037 | 0.7957 | 32 | 0.6538 |
|  |  | PCL-Alg10p | Proteins | *k* = 40.06  (38.14, 53.98) | 1156354 | 0.1345 | 32 | 3.894 |
|  |  |  | Lipids | *k* = 10.93  (9.061, 12.79) | 64192 | 0.432 | 32 | 0.9175 |
|  |  | cPCL-Alg6 | Proteins | *k* = 25.43  (17.19, 33.67) | 271555 | 0.3602 | 32 | 4 |
|  |  |  | Lipids | *k* = 7.101  (4.668, 9.534) | 23690 | 0.3949 | 32 | 1.181 |
|  |  | cPCL-Alg10 | Proteins | *k* = 39.96  (36.99, 42.93) | 35392 | 0.9223 | 32 | 1.444 |
|  |  |  | Lipids | *k* = 8.519  (7.892, 9.145) | 1572 | 0.9337 | 32 | 0.3043 |
|  |  | cPCL-Alg10p | Proteins | *k* = 44.5  (38.57, 50.43) | 648266 | 0.09657 | 32 | 2.916 |
|  |  |  | Lipids | *k* = 2.555  (2.106, 3.003) | 3704 | 0.6405 | 32 | 0.2204 |
| Higuchi  [37] | *F*(*t*) = 100 × (1−C × exp ^(−^*^k^* ^×^ *^t^*^)^) | PCL | Proteins | C = 0.9873  (0.8484, 1.125)  *k* = 2.597  (1.682,4.343) | 4126 | 0.826 | 31 | C  0.06646  *k*  0.4488 |
|  |  |  | Lipids | C = 0.9978  (0.8707, 1.125)  *k* = 3.956  (2.579, +∞) | 3587 | 0.8889 | 31 | C  0.06209  *k*  0.8755 |
|  |  | PCL-Alg6 | Proteins | C = 0.9919  (0.8801, 1.103)  *k* = 2.806  (2.014, 4.191) | 2736 | 0.9006 | 31 | C  0.05416  *k*  0.4107 |
|  |  |  | Lipids | C = 0.9368  (0.8187, 1.056)  *k* = 1.013  (0.7262, 1.381) | 1290 | 0.8759 | 31 | C  0.03611  *k*  0.02139 |
|  |  | PCL-Alg10 | Proteins | C = 0.9192  (0.7823, 1.057)  *k* = 0.9425  (0.6074, 1.390) | 3830 | 0.8759 | 31 | C  0.06065  *k*  0.1395 |
|  |  |  | Lipids | C = 0.8785  (0.7924, 0.9635)  *k* = 0.1636  (0.1063, 0.2274) | 2685 | 0.922 | 31 | C  0.03611  *k*  0.02139 |
|  |  | PCL-Alg10p | Proteins | C = -2.013  (−2.717, −1.348)  *k* = -0.003833  (−0.006036, −0.001721) | 960609 | 0.281 | 31 | C  0.3518  *k* 0.001099 |
|  |  |  | Lipids | C = 0.1672  (0.6326, 1.337)  *k* = 0.102  (0.03750, 0.3555) | 51190 | 0.5471 | 31 | C  0.1672  *k*  0.06062 |
|  |  | cPCL-Alg6 | Proteins | C = -0.06008  (−0.4161, −1.103)  *k* = -0.03301  (−114.9, + ∞) | 371836 | 0.124 | 31 | C  0.1589  *k*  0.0296 |
|  |  |  | Lipids | C = 1.005  (0.8113, 1.206)  *k* = 0.0155  (0.006715, 0.02832) | 22957 | 0.4137 | 31 | C  0.09482  *k* 0.004879 |
|  |  | cPCL-Alg10 | Proteins | C = −0.3164  (−0.6488, −0.07990)  *k* = −0.02346  (−0.03818, -0.01378) | 247087 | 0.4573 | 31 | C  0.1747  *k* 0.006711 |
|  |  |  | Lipids | C = 0.9368  (0.8187, 1.056)  *k* = 1.013  (0.7262, 1.381) | 1290 | 0.8759 | 31 | C  0.03611  *k*  0.02139 |
|  |  | cPCL-Alg10p | Proteins | C = −1.892  (−2.293, −1.505)  *k* = −0.004127  (−0.005422, −0.002877) | 317038 | 0.5582 | 31 | C  0.2004  *k* 0.0006508 |
|  |  |  | Lipids | C = 0.9651  (0.9104, 1.020)  *k* = 0.002239  (0.001528, 0.003018) | 4112 | 0.6009 | 31 | C  0.02675  *k* 0.0003557 |
| Peppas– Sahlin | *F*(*t*) = *k_1_* × *t^m^* + *k_2_* × *t*^(2 ×^ *^m^*^)^ | PCL | Proteins | *k_1_* = ~  *k_2_* = ~  *m* = ~ | 714.2 | 0.9699 | 30 | *k_1_* ~  *k_2_* ~  m ~ |
|  |  |  | Lipids | *k_1_* = ~  *k_2_* = ~  *m* = ~ | 3117 | 0.9034 | 30 | *k_1_* ~  *k_2_* ~  m ~ |
|  |  | PCL-Alg6 | Proteins | *k_1_* = ~  *k_2_* = ~  *m* = ~ | 1547 | 0.9438 | 30 | *k_1_* ~  *k_2_* ~  m ~ |
|  |  |  | Lipids | *k_1_* = ~  *k_2_* = ~  *m* = ~ | 933.1 | 0.9529 | 19 | *k_1_* ~  *k_2_* ~  m ~ |
|  |  | PCL-Alg10 | Proteins | *k_1_* = ~  *k_2_* = ~  *m* = ~ | 2173 | 0.9296 | 30 | *k_1_* ~  *k_2_* ~  m ~ |
|  |  |  | Lipids | *k_1_* = ~  *k_2_* = ~  *m* = ~ | 849.8 | 0.9753 | 30 | *k_1_* ~  *k_2_* ~  m ~ |
|  |  | PCL-Alg10p | Proteins | *k_1_* = ~  *k_2_* = ~  *m* = ~ | 455849 | 0.6588 | 31 | *k_1_* ~  *k_2_* ~  m ~ |
|  |  |  | Lipids | *k_1_* = ~  *k_2_* = ~  *m* = ~ | 42712 | 0.6221 | 31 | *k_1_* ~  *k_2_* ~  m ~ |
|  |  | cPCL-Alg6 | Proteins | *k_1_* = −143.6  *k_2_* = 158.4  *m* = 0.1229 | 268899 | 0.3665 | 31 | *k_1_* 580.5  *k_2_* 554.9  m 0.221 |
|  |  |  | Lipids | *k_1_* = −19.88  *k_2_* = 18.97  *m* = 0.2076 | 23030 | 0.4118 | 31 | *k_1_* 62.63  *k_2_* 53.1  m 0.2226 |
|  |  | cPCL-Alg10 | Proteins | *k_1_* = −372.9  *k_2_* = 376.2  *m* = 0.1041 | 21392 | 0.953 | 31 | *k_1_* 210.2  *k_2_* 203.4  m 0.0324 |
|  |  |  | Lipids | *k_1_* = −18.5  *k_2_* = 18.25  *m* = 0.2251 | 668.5 | 0.9718 | 31 | *k_1_* 9.431  *k_2_* 7.768  m 0.0342 |
|  |  | cPCL-Alg10p | Proteins | *k_1_* = ~  *k_2_* = ~  *m* = ~ | 57047 | 0.9205 | 31 | *k_1_* ~  *k_2_* ~  m ~ |
|  |  |  | Lipids | *k_1_* = −15.27  *k_2_* = 13.88  *m* = 0.154 | 3570 | 0.6535 | 31 | *k_1_* 23.63  *k_2_* 20.32  m 0.0918 |
| Zero-order | *F*(*t*) = *k* × *t* | PCL | Proteins | *k* = 3.038  (2.182, 3.895) | 91138 | −2.842 | 32 | 0.4204 |
|  |  |  | Lipids | *k* = 3.327  (2.800, 3.853) | 137561 | −3.262 | 32 | 0.5165 |
|  |  | PCL-Alg6 | Proteins | *k* = 3.195  (2.271, 4.119) | 106126 | −2.856 | 32 | 0.4537 |
|  |  |  | Lipids | *k* = 3.18  (2.152, 4.209) | 55198 | −1.784 | 21 | 0.4947 |
|  |  | PCL-Alg10 | Proteins | *k* = 3.187  (2.382, 3.993) | 80652 | −1.613 | 32 | 0.3955 |
|  |  |  | Lipids | *k* = 2.863  (2.388, 3.338) | 28034 | 0.186 | 32 | 0.2332 |
|  |  | PCL-Alg10p | Proteins | *k* = 3.102  (2.206, 3.998) | 2420359 | −0.8117 | 32 | 0.4404 |
|  |  |  | Lipids | *k* = 0.755  (0.5518, 0.9582) | 124514 | −0.1017 | 32 | 0.09988 |
|  |  | cPCL-Alg6 | Proteins | *k* = 3.14  (1.966, 4.313) | 320842 | 0.2441 | 32 | 0.5697 |
|  |  |  | Lipids | *k* = 0.9166  (0.5911, 1.242) | 24702 | 0.3691 | 32 | 0.1581 |
|  |  | cPCL-Alg10 | Proteins | *k* = 4.958  (4.163, 5.754) | 147388 | 0.6763 | 32 | 0.3861 |
|  |  |  | Lipids | *k* = 1.103  (0.9943, 1.211) | 2747 | 0.8841 | 32 | 0.05271 |
|  |  | cPCL-Alg10p | Proteins | *k* = 3.058  (2.309, 3.806) | 1688830 | -1.354 | 32 | 0.3678 |
|  |  |  | Lipids | *k* = 1924  (0.1525, 0.2322) | 4788 | 0.5353 | 32 | 0.019959 |


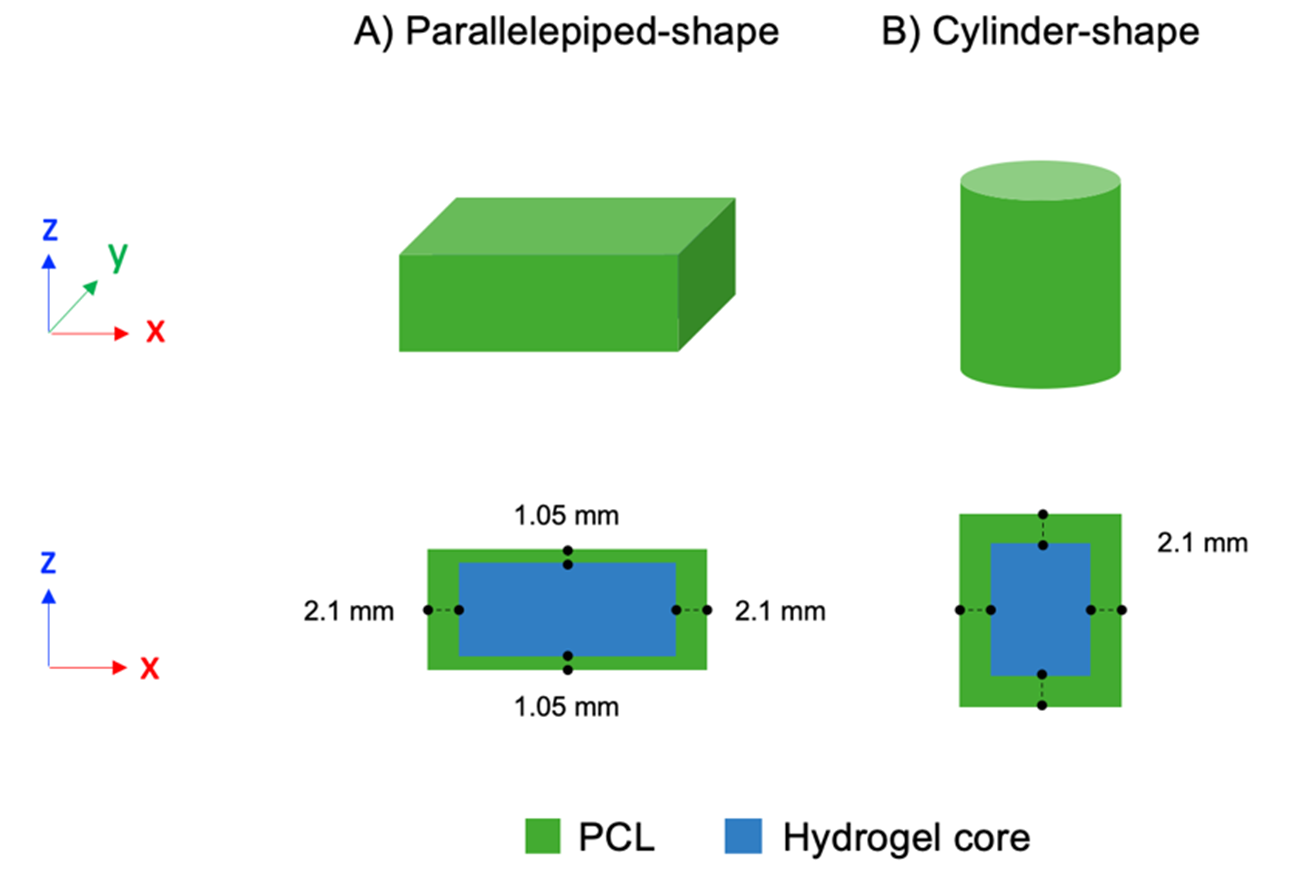


**Figure S3.** Lyosecretome alginate core position inside the PCL scaffold structure. For the parallelepiped-shaped structure (**A**) the distance from the alginate core to the outside is not the same in XYZ directions. In the cylindrical-shape structure (**B**), there is the same distance from the alginate core to the outside in all XYZ directions.

References

1. Caccavo, D. An overview on the mathematical modeling of hydrogels’ behavior for drug delivery systems. *Int. J. Pharm.* **2019**, *560*, 175–190, doi:10.1016/j.ijpharm.2019.01.076.
